# Supplementary material for: Detection of risk for depression among adolescents in diverse global settings: protocol for the IDEA qualitative study in Brazil, Nepal, Nigeria and the UK
Source: BMJ Open. 2020 Jul 28;10(7):e034335. doi: 10.1136/bmjopen-2019-034335 (PMC7389769; doi:10.1136/bmjopen-2019-034335)
Supplement: Supplementary data [file bmjopen-2019-034335supp001.pdf]

Protocol for the IDEA qualitative study on risk for adolescent depression

Appendix-1

Study settings: Country descriptions

Brazil

Brazil is an upper middle-income country representing the growing number of countries with rapidly developing economies and urbanization, collectively known as the BRICS nations (Brazil, Russia, India, China, and South Africa). Two out of every three adolescents worldwide live in a BRICS country. Brazil has a total population of 210 million people; and the prevalence of major depressive disorder has been estimated to be around 9.4 percent among adults, being one of the highest rates in Latin America.[1] The health and education systems are characterized by a mix of public and private services. Overall, the education system is divided into preschool, fundamental school (nine years) and high school (three additional years). The public Unified Health System provides universal health coverage free of charge. Mental health care is provided at multiple levels of complexity, including psychosocial centers (2,462 units in 2017, with only 229 specialized to the care of children and adolescents).[2] The social services system is organized based on the specificities of each territory. The child care council is the organization responsible for ensuring the rights of children and adolescents.[3]

In Brazil, the qualitative study will be implemented by a team from the Universidade Federal do Rio Grande do Sul. Data collection efforts will be led by five researchers from IDEA Brazil team: one psychologist, two psychology undergraduate students, and one with social communication training (all females); and two medical doctors (both males), which includes an IDEA principal investigator. The recruitment process will utilize connections of the IDEA Brazil team to local, relevant, institutions. The key informant interviews (KII) will take place in private and convenient locations for each participant, and the focus group discussions (FGD) will be held in Hospital de Clínicas de Porto Alegre.

Nepal

Nepal is one of the poorest countries in the world and represents conditions of adolescents living in the least-developed nations. Nepal also is representative of the large number of adolescents living in humanitarian settings. Adolescents in Nepal today grew up during a decade-long civil war, and many have been impacted by repeated environmental disasters including earthquakes, flooding, and landslides. More than 20 percent of the national population have at least one mental health problem in Nepal.[4] Adolescents comprise 24 percent of the total population of Nepal, and 14 percent of this population have one or more psychosocial problems.[5, 6] Known risk factors contributing to depression include political conflict, caste-based disparities, earthquake, urban habitation, domestic violence, poverty, and other social factors.[7-9] There is a single outpatient child and adolescent psychiatry clinic and no corresponding inpatient facilities. The National Mental Health Policy was developed in Nepal in 1996 to integrate mental health into primary health care services.[10] The Ministry of Health and Population has adapted the World Health Organization's (WHO) Mental Health Gap Action Programme (mhGAP), developed a community mental health care package training curricula to non-specialized mental health service providers, revised psychotropic medications in the

essential drug list, included four priority Mental Neurological and Substance use disorders (depression, psychosis, epilepsy and alcohol use disorder) in basic health service package and revised the National Adolescent Health and Development Strategy.[11] The Ministry of Health & Population has been drafting the National Mental Health Strategy and Action Plan in an attempt to reinforce integration of mental health into primary care with a focused strategy on child and adolescent mental health which was not included in the National Mental Health Policy 1996. The Ministry of Education has piloted the introduction of school nurses as an initiative to improve child and adolescent health in selected regions of the country.

The data collection efforts in Nepal will be implemented by the Transcultural Psychosocial Organization Nepal (TPO Nepal), one of Nepal's leading psychosocial organizations for providing clinical care and conducting mental health research. TPO interviewers and FGD facilitators will include two female researchers with graduate level training in Public Health and one male researcher with graduate training in management studies, under the guidance of a senior TPO director and researcher, who is an IDEA study Co-investigator. Recruitment for the study will be facilitated by reaching out to institutions offering adolescent mental health services in Kathmandu district in Nepal. KIIs will be conducted in private locations at health facilities, schools, stakeholder offices, TPO Nepal offices, Ministry offices, etc. The FGDs will each be conducted at TPO offices. KIIs and FGDs will be primarily conducted in Nepali & English.

Nigeria

Nigeria is a lower-income country and the most populous country in Africa. The experiences of adolescents in Nigeria reflect the rapid development of African economies as well as chronic exposure to political violence, community violence, and high rates of infectious diseases, including HIV/AIDS. In Nigeria, adolescents (age 10-19 years) make up approximately 41 million, or 23 percent of the total population of 180 million.[12] In 2015, the WHO estimated the prevalence of depression to be approximately 3.9 percent in the general population.[13] However, nationally representative research has indicated that up to 10 percent of the Nigerian school adolescents to have clinical depression.[14] With over 15 million adolescents in its secondary schools, Nigeria has an estimated 1.5 million depressed school adolescents in need of intervention.[15] Mental health remains under-prioritized, and is unaddressed in the National Health Policy and National Strategic Health Development Plan (2010-2015), as well as in educational and criminal justice policies.[16] Similarly, child and adolescent mental health are not mentioned in the National Child Health Policy. Additionally, the School Health Policy launched by the Federal Government in 2006 did not have provisions for mental health.[17] There are no government-run health facilities specifically for children and adolescents, and less than 20 percent of primary and secondary schools have activities that promote mental health.[18] Social and health risk factors for poor mental health, particularly for children and adolescents, include high levels of poverty and child malnutrition, as well as the prevalence of child labor and under-age marriage.[19] In Lagos State, where the IDEA project will be implemented, there are over one million adolescents in its nearly 3,000 secondary schools.[20]

In Nigeria, the study is managed by the Lagos State University, College of Medicine, Department of Behavioral Medicine. Data collection efforts will be led by a team of two female doctors, a consultant psychiatrist, and a medical officer under guidance of a senior professor, who is an IDEA project co-investigator. Recruitment will be through flyers distributed to social

workers, health care providers and the teachers in both public and private schools in Lagos. Letters will be written to the permanent secretaries of the ministries of health, education, social services and the health service commission requesting the nomination of civil servants for policy-maker interviews of the respective ministries. Interviews will be done at the convenience of the participant, and the timing and the location will be flexible. Interviews can be held at the office of the researchers or at the offices of the person being interviewed. The FGDs will be held at Department of Behavioural Medicine at The Lagos State University Teaching Hospital and at the Child and Adolescent Mental Health Service of The Federal Neuropsychiatry Hospital Oshodi Annex.

United Kingdom

The UK is the study setting representative of adolescents living in highly resourced regions such as Western Europe and North America. Adolescents aged 10-19 years old comprise approximately 12 percent of the total population of the UK, of whom 17 percent (2.3 million children) are living in persistent poverty, and 18 percent do not attain a baseline level of proficiency in reading to enable them to participate effectively in activities requiring literacy.[21-23] In the UK, one in seven 11-16 year-olds has at least one mental illness, however, only 25 percent of these receive support from the National Health Service (NHS).[24] The NHS Long Term Plan[25] highlights prevention as a national priority for mental health. Members of the UK also have their own mental health policies, strategies, or programs for the general population. England, Scotland, and Wales have national mental health strategies and policies that support those with mental illnesses by promoting anti-stigma campaigns and recommendations for health providers. In the UK, Child and Adolescent Mental Health Services (CAMHS) are provided by the NHS and cover community and hospital-based care up to 17 years of age. Countries within the UK each feature some variant of a task force or program responsible for improving the quality and accessibility of health care for children and young people, aiming to increase government funding, decrease waiting times, improve school counseling services, and increase social services provision to support child development.[24, 26]

In the UK, the data collection efforts are implemented by King’s College London. The primary data collector is a female social scientist with graduate training in nursing and psychology, with experience in conducting qualitative research. The UK activities are guided by a co-investigator of the IDEA project. Participants will be recruited using an opportunistic sampling method, with stakeholder specific adverts being circulated via professional networks, social media, and placed in key locations in the community, including local NHS hospitals. The KIIs will take place at either one of the King’s College London campuses, stakeholder’s office spaces where appropriate, or over the telephone.

References

1. Andrade, L.H., Wang, Y.P., Andreoni, S., et al. Mental disorders in megacities: findings from the Sao Paulo megacity mental health survey, Brazil. *PLoS One*, 2012. 7(2): p. e31879.
2. Trape, T.L. and Campos, R.O. The mental health care model in Brazil: analyses of the funding, governance processes, and mechanisms of assessment. *Rev Saude Publica*, 2017. 51(0): p. 19.

3. Ministry of Social Development and Fight Against Hunger. 2011.
4. Risal, A., Manandhar, K., Linde, M., et al. Anxiety and depression in Nepal: prevalence, comorbidity and associations. *BMC Psychiatry*, 2016. 16(1): p. 102.
5. Adhikari R.P., Upadhaya N., Pokhrel R., et al. Health and social vulnerability of adolescents in Nepal. *SM J Public Health and Epi*, 2016. 2(3): p. 1032.
6. Central Bureau of Statistics, National Population and Housing Census 2011,. 2011, Government of Nepal: Kathmandu, Nepal.
7. Upadhaya, N., Tize, C., Adhikari, R., et al. Geographies of adolescent distress: A need for a community-based psychosocial care system in Nepal. *Intervention*, 2018. 17.
8. Kohrt, B.A., Hruschka, D.J., Worthman, C.M., et al. Political violence and mental health in Nepal: prospective study. *Br J Psychiatry*, 2012. 201(4): p. 268-75.
9. Kohrt, B.A., Jordans, M.J.D., Tol, W.A., et al. Social ecology of child soldiers: child, family, and community determinants of mental health, psychosocial well-being, and reintegration in Nepal. *Transcult Psychiatry*, 2010. 47(5): p. 727-753.
10. Luitel, N.P., Jordans, M.J., Adhikari, A., et al. Mental health care in Nepal: current situation and challenges for development of a district mental health care plan. *Confl health*, 2015. 9: p. 3-3.
11. Kohrt, B.A., Mutamba, B.B., Luitel, N.P., et al. How competent are non-specialists trained to integrate mental health services in primary care? Global health perspectives from Uganda, Liberia, and Nepal. *Int Rev Psychiatry*, 2018. 30(6): p. 182-198.
12. Affairs, U.N.D.o.E.a.S., World Population Prospects 2019.
13. World Health Organization, Depression and other common mental disorders: Global health estimates. 2017, World Health Organization: Geneva.
14. Adewuya, A.O., Ola, B.A. and Aloba, O.O. Prevalence of major depressive disorders and a validation of the Beck Depression Inventory among Nigerian adolescents. *Eur Child Adolesc Psychiatry*, 2007. 16(5): p. 287-92.
15. National Population Commission (NPC) [Nigeria] and ICF International, Nigeria Demographic and Health Survey 2013. 2014, NPC and ICF International: Abuja, Nigeria, and Rockville, Maryland.
16. Abdulmalik, J., Kola, L. and Gureje, O. Mental health system governance in Nigeria: challenges, opportunities and strategies for improvement. *Global Mental Health*, 2016. 3: p. e9-e9.
17. Federal Ministry of Education Nigeria, National School Health Policy. 2006: Abuja, Nigeria.
18. World health Organization, M.o.H.N., WHO-AIMS report on Mental Health System in Nigeria,. 2006: Ibadan, Nigeria.
19. Atilola, O., Ayinde, O.O., Emedoh, C.T., et al. State of the Nigerian child - neglect of child and adolescent mental health: a review. *Paediatr Int Child Health*, 2015. 35(2): p. 135-43.
20. Education Sector Support Programme in Nigeria (ESSPIN), Lagos Private School Census 2010-2011 Report. 2011, ESSPIN: Lagos, Nigeria.
21. Hagell, A., Coleman, J. and Brooks, F., Key Data on Adolescence 2015. 2015, Association for Young People's Health: London.
22. Social Metrics Commission, Measuring poverty 2019. 2019: United Kingdom.
23. Organization for Economic Cooperation and Development, United Kingdom Country Note – Results from PISA 2015. 2019.
24. National Health Service, Mental Health of Children and Young People in England. 2018.
25. National Health Service, The NHS Long Term Plan. 2019.
26. Health and Social Care, Children and Young People's Mental Health Taskforce: Delivery Plan. 2018, Government of Scotland.
