# Supplementary material for: Detection of risk for depression among adolescents in diverse global settings: protocol for the IDEA qualitative study in Brazil, Nepal, Nigeria and the UK
Source: BMJ Open. 2020 Jul 28;10(7):e034335. doi: 10.1136/bmjopen-2019-034335 (PMC7389769; doi:10.1136/bmjopen-2019-034335)
Supplement: Author's manuscript [file bmjopen-2019-034335.draft_revisions.pdf]

BMJ Open

BMJ Open is committed to open peer review. As part of this commitment we make the peer review history of every article we publish publicly available.

When an article is published we post the peer reviewers' comments and the authors' responses online. We also post the versions of the paper that were used during peer review. These are the versions that the peer review comments apply to.

The versions of the paper that follow are the versions that were submitted during the peer review process. They are not the versions of record or the final published versions. They should not be cited or distributed as the published version of this manuscript.

BMJ Open is an open access journal and the full, final, typeset and author-corrected version of record of the manuscript is available on our site with no access controls, subscription charges or pay-per-view fees (http://bmjopen.bmj.com).

If you have any questions on BMJ Open's open peer review process please email info.bmjopen@bmj.com

BMJ Open

Detection of risk for depression among adolescents in diverse global settings: Protocol for the IDEA qualitative study in Brazil, Nepal, Nigeria and the United Kingdom

Journal:	*BMJ Open*
Manuscript ID	bmjopen-2019-034335
Article Type:	Protocol
Date Submitted by the Author:	15-Sep-2019

Complete List of Authors:	Wahid, Syed; George Washington University, Division of Global Mental Health<br>Pedersen, Gloria; George Washington University,<br>Ottman, Katherine; George Washington University, Division of Global Mental Health<br>Burgess, Abigail; King's College London, Psychology and Neuroscience<br>Gautam, Kamal; Transcultural Psychosocial Organization Nepal<br>Martini, Thais; Universidade Federal do Rio Grande do Sul, Department of Psychiatry<br>Viduani, Anna; Universidade Federal do Rio Grande do Sul, Department of Psychiatry<br>Momodu, Olufisayo; Lagos Island General Hospital, Department of Psychiatry<br>Lam, Crystal; George Washington University, Department of Global Health<br>Fisher , Helen; King's College London, SGDP Centre<br>Kieling, Christian; Universidade Federal do Rio Grande do Sul, Department of Psychiatry; Hospital de Clínicas de Porto Alegre, Child & Adolescent Psychiatry Division<br>Adewuya, Abiodun O; Lagos State University College of Medicine,<br>Mondelli, Valeria; King's College London<br>Kohrt, Brandon A.; Duke Univ; George Washington University, Division of Global Mental Health

Keywords:	adolescent, depression, risk factors, protective factors, qualitative

SCHOLARONE™
Manuscripts

I, the Submitting Author has the right to grant and does grant on behalf of all authors of the Work (as defined in the below author licence), an exclusive licence and/or a non-exclusive licence for contributions from authors who are: i) UK Crown employees; ii) where BMJ has agreed a CC-BY licence shall apply, and/or iii) in accordance with the terms applicable for US Federal Government officers or employees acting as part of their official duties; on a worldwide, perpetual, irrevocable, royalty-free basis to BMJ Publishing Group Ltd ("BMJ") its licensees and where the relevant Journal is co-owned by BMJ to the co-owners of the Journal, to publish the Work in this journal and any other BMJ products and to exploit all rights, as set out in our licence.

The Submitting Author accepts and understands that any supply made under these terms is made by BMJ to the Submitting Author unless you are acting as an employee on behalf of your employer or a postgraduate student of an affiliated institution which is paying any applicable article publishing charge ("APC") for Open Access articles. Where the Submitting Author wishes to make the Work available on an Open Access basis (and intends to pay the relevant APC), the terms of reuse of such Open Access shall be governed by a Creative Commons licence – details of these licences and which Creative Commons licence will apply to this Work are set out in our licence referred to above.

Other than as permitted in any relevant BMJ Author's Self Archiving Policies, I confirm this Work has not been accepted for publication elsewhere, is not being considered for publication elsewhere and does not duplicate material already published. I confirm all authors consent to publication of this Work and authorise the granting of this licence.

For peer review only

Title: Detection of risk for depression among adolescents in diverse global settings: Protocol for the IDEA qualitative study in Brazil, Nepal, Nigeria and the United Kingdom

Running Head: Protocol for the IDEA qualitative study on risk for adolescent depression

Key words: adolescent; depression; risk factors, protective factors; qualitative; framework analysis; delphi method; theory of change;

Authors:

Syed Shabab Wahid
Division of Global Mental Health, George Washington University, Washington DC, United States.

Gloria A. Pedersen
Division of Global Mental Health, George Washington University, Washington DC, United States.

Katherine Ottman
Division of Global Mental Health, George Washington University, Washington DC, United States.

Abigail Burgess
Social, Genetic & Developmental Psychiatry Centre, Institute of Psychiatry, Psychology and Neuroscience, King's College London, London, United Kingdom.

Kamal Gautam
Transcultural Psychosocial Organization Nepal (TPO Nepal), Baluwatar, Kathmandu, Nepal

Thais Martini
Department of Psychiatry, Universidade Federal do Rio Grande do Sul, Porto Alegre, RS, Brazil.

Anna Viduani
Department of Psychiatry, Universidade Federal do Rio Grande do Sul, Porto Alegre, RS, Brazil.

Olufisayo Momodu
Department of Psychiatry, Lagos Island General Hospital, Lagos, Nigeria.

Crystal Lam
Department of Global Health, George Washington University, Washington DC, United States.

Helen L. Fisher*
Social, Genetic & Developmental Psychiatry Centre, Institute of Psychiatry, Psychology & Neuroscience, King's College London, London, United Kingdom.

For peer review only

For peer review only - http://bmjopen.bmj.com/site/about/guidelines.xhtml

Christian Keiling
Department of Psychiatry, Universidade Federal do Rio Grande do Sul and Child & Adolescent Psychiatry Division, Hospital de Clínicas de Porto Alegre, Porto Alegre, RS, Brazil.

Abiodun Adewuya
Department of Behavioural Medicine, Lagos State University College of Medicine, Lagos, Nigeria.

Valeria Mondelli
Department of Psychological Medicine, Institute of Psychiatry, Psychology & Neuroscience, King's College London, London, United Kingdom.

Brandon A. Kohrt
Division of Global Mental Health, George Washington University, Washington DC, United States.

***Corresponding author**:
Helen L. Fisher*
Social, Genetic & Developmental Psychiatry Centre, Institute of Psychiatry, Psychology & Neuroscience, King's College London, London, United Kingdom. helen.2.fisher@kcl.ac.uk Tel: 020-7848-5430

Author contributions:
BAK conceived the paper and was in charge of overall direction and planning. SSW, GAP, KO, AB, KG, TM, AV, OM, CL wrote the manuscript with input from all the authors. CK, HLF, AA, VM and BAK provided overall technical review, critical revision, and final approval for publication.

Total words: 5,007 words (BMJ word limit: 4,000)
This study exceeds the BMJ Open word limit for research protocols. We are requesting the editor to accept the larger word count based on two considerations: (1) the study has four operational sites and thus requires background information on each site in the "Study Setting" section; and (2) the methods section of the study had to be significantly broader to appropriately address the 3 distinct methods (Delphi, Theory of Change, and qualitative interviews) being utilized.

Funding
MQ Transforming Mental Health Charity, Brighter Futures grant named "Identifying Depression Early in Adolescence", Grant #MQBF/1 IDEA. Additional support was provided by the UK Medical Research Council (MC_PC_MR/R019460/1) and the Academy of Medical Sciences (GCRFNG\100281) under the Global Challenges Research Fund. HLF is supported by a British Academy Mid-Career Fellowship (MD\170005). CK has received support from Brazilian governmental research funding agencies (Conselho Nacional de Desenvolvimento Científico e Tecnológico [CNPq], Coordenação de Aperfeiçoamento de Pessoal de Nível Superior [CAPES], and Fundação de Amparo à Pesquisa do Estado do Rio Grande do Sul [Fapergs]). VM has been

For peer review only - http://bmjopen.bmj.com/site/about/guidelines.xhtml

supported by the National Institute for Health Research (NIHR) Mental Health Biomedical Research Centre at South London and Maudsley NHS Foundation Trust and King's College London. The views expressed are those of the authors and not necessarily those of the National Health Service, the NIHR, or the Department of Health. BAK is supported by the US National Institute of Mental Health (K01MH104310, R21MH111280).

Study Dates

The operational dates of the IDEA study are from November 2018 to November 2021.

Competing interests

We declare that we have no competing interests.

For peer review only

For peer review only - http://bmjopen.bmj.com/site/about/guidelines.xhtml

ABSTRACT

Introduction

Globally, depression is a leading cause of disability among adolescents, and suicide rates are increasing among youth. Treatment alone is insufficient to address the issue. Early identification and prevention efforts are necessary to reduce morbidity and mortality. The Identifying Depression Early in Adolescence (IDEA) consortium is developing risk detection strategies that incorporate biological, psychological, and social factors that can be evaluated in diverse global populations. In addition to epidemiological and neuroscience research, the IDEA consortium is conducting a qualitative study to explore three domains of inquiry: (1) cultural heterogeneity of biopsychosocial risk factors and lived experience of adolescent depression in low- and middle-income countries (LMIC); (2) the feasibility, acceptability, and ethics of a risk calculator tool for adolescent depression that can be used in LMIC and high-income countries; and (3) capacity for biological research into biomarkers for depression risk among adolescents in LMIC. This a multi-site qualitative study being conducted in Brazil, Nepal, Nigeria, and the United Kingdom.

Methods and analysis

A systematic set of qualitative methods will be utilized in this study. The Delphi method, Theory of Change (ToC) workshops, key-informant interviews, and focus group discussions will be used to elicit perspectives on the study topics from a broad range of stakeholders (adolescents, parents, policymakers, teachers, health service providers, social workers, and experts). Delphi panelists will participate in 3 survey rounds to generate consensus through facilitated feedback. Stakeholders will create ToC models via facilitated workshops in the LMIC sites. The framework approach will be used to analyze data from the study.

Ethics and dissemination

Ethical approvals were received from the Ethics Review Board of George Washington University and from site-specific institutions in Brazil, Nepal, Nigeria, and the UK. The findings generated from this study will be reported in highly accessed, peer-reviewed, scientific and health policy journals.

ARTICLE SUMMARY

Strengths and limitations of this study

- This study uses the Delphi method, the Theory of Change approach, key-informant interviews and focus group discussion methods in multiple settings varied by culture, economic status, and risk factors to investigate adolescent depression
- The multi-site approach will generate new knowledge on context-specific and universal pathways of adolescent depression manifestation, identification, and prevention
- Perspectives will be elicited from a broad range of stakeholders including adolescents and parents, teachers, health service providers, social workers, and policymakers

For peer review only - http://bmjopen.bmj.com/site/about/guidelines.xhtml

INTRODUCTION

Globally, depression is a leading cause of illness and disability among adolescents aged 10-19 years old.[1] The presence of depression also increases the risk of suicide, which is the third leading cause of death in this population.[1] As the incidence of depression peaks in adolescence, and often remains undiagnosed, the negative consequences of depression persist as a chronic condition throughout the life course.[2] Major depressive disorder, the most commonly diagnosed form of depression, has a lifetime prevalence of 11 percent representing a major cause of disability across the world.[3]

On a global scale, treatment alone is insufficient to address this problem. Limited efficacy of available interventions along with the limited availability and low-quality of mental health services in many parts of the world persist as significant barriers.[1, 4, 5] Therefore, increased identification of depression early in adolescence and administering preventive strategies become highly salient in addressing this global burden.[5] Understanding the biopsychosocial risk factors that can predict the onset of depression, and the protective factors which can inform measures for preventing its manifestation and severity, are important steps towards achieving this goal.

Research on depression among adolescents in Western Educated Industrialized Rich and Democratic (WEIRD) countries is insufficient to understand the full scale of the problem.[6] Identification and prevention measures need to account for the heterogeneity of risk and protective factors and signs and symptoms of depression, as they manifest across cultures and countries.[7] Therefore, investigating depression across multiple global settings can provide clearer insight into the universal and context-specific risk and protective factors.

Currently, there are three major gaps in global research to address prevention and early identification among adolescents. First, there is a need for context-specific understandings of mental health, adolescent experiences, and risk and protective factors. Cultural conceptions of depression or adolescence vary in their meaning and social implications across populations and context. Accordingly, discerning such factors are crucial in informing culturally-sensitive depression identification and prevention efforts.

Second, despite a wide range of research on depression, there is a lack of feasible and acceptable tools to determine the risk of depression among adolescents before the disorder develops. Risk scores are important in other fields of medicine to determine when and how to intervene and how best to allocate resources, e.g., risk determination and treatment provision for cardiovascular disease, diabetes, and cancer.[8-10] Adopting similar tenets to develop a risk calculator for adolescent depression could have substantial public health benefit.

Third, the ethical and institutional policies for research with adolescents vary widely in low- and middle-income countries (LMIC). Specifically, ethical policies are often lacking for biological psychiatry research, which is needed to shed light on mechanisms and risk markers for adolescent depression.[11] Biological markers may be an important contributor to risk calculation for depression and to evaluate efficacy of prevention efforts.[12] Therefore, assessing the feasibility of implementing biological psychiatry research and determining its ethical and cultural acceptability is critical to identify necessary infrastructural and policy recommendations to increase LMIC capacity for conducting such studies.

To address these three gaps, this article presents the protocol of a global, multi-site, qualitative study which will inform the development of successful adolescent depression

For peer review only - http://bmjopen.bmj.com/site/about/guidelines.xhtml

identification and prevention initiatives. First, in our qualitative study, we will investigate the cultural and contextual perceptions and considerations of identifying adolescent depression, including risk and protective factors, and depression prevention interventions. Second, we will explore the feasibility, acceptability, and utility of a risk calculator for depression in adolescence. Third, we will investigate the feasibility of conducting biological psychiatry research in LMIC settings. To achieve this, a diverse set of qualitative methods will be utilized in four countries: Brazil, Nepal, Nigeria, and the United Kingdom (UK), representing high-, middle- and low-income settings.

OBJECTIVES

Identifying Depression Early in Adolescence (IDEA) Research Consortium

This qualitative study is part of a larger integrated research portfolio being implemented by the Identifying Depression Early in Adolescence (IDEA) research consortium (https://www.mqmentalhealth.org/research/profiles/identifying-depression-early-in-adolescence). The consortium comprises psychiatrists, epidemiologists, neuroscientists, and anthropologists from Brazil, Nepal, Nigeria, the UK, and United States conducting multi-disciplinary research on adolescent depression identification and prevention. The consortium was established with the support of the MQ charity Brighter Futures initiative. The operational dates of the IDEA study are from November, 2018 to November 2021.

General Objective of Multi-Component Qualitative Study:

The objective of the current study is to qualitatively explore key topics that will compliment epidemiological and biological psychiatry research being conducted through IDEA. For this multi-component qualitative study, we will consult with adolescents, parents, health care providers, social workers, educators, policymakers and other key stakeholders in Brazil, Nepal, Nigeria and the UK, as well as global experts, about the experience of adolescent depression cross-culturally, the use of a risk calculator for depression during adolescence, and conducting biological psychiatry research with adolescents in LMIC.

Specific Objectives:

1. The qualitative component of IDEA will compare cultural and contextual differences in depression, adolescent experiences, health systems, and health policy across Brazil, Nepal, Nigeria, and the UK. Additionally, information will be collected from an expert panel on risk factors and detection approaches that are specific for adolescent depression and feasible for implementation in LMIC.
2. A risk assessment tool for depression in adolescence is currently being developed via predictive modeling using cohort data from Brazil, Nigeria, Nepal and the UK.[5] The tool will determine future risk of depression and is derived from research that classifies risk based on the presence of biological, psychological, and social risk factors.[13] This qualitative study will explore the feasibility, acceptability, utility and the ethical implications of such a tool.
3. The IDEA research includes biological psychiatry studies with functional neuroimaging and inflammation pathways. The qualitative work will also explore feasibility, acceptability, and

For peer review only - http://bmjopen.bmj.com/site/about/guidelines.xhtml

ethics related to expanding biological psychiatry research in LMIC and incorporating biological markers to evaluate risk of the onset of depression during adolescence.

METHODS
To conduct this study, three methodologies will be used: a Delphi activity, theory of change workshops, and qualitative interviews including both key informant interviews (KII) and focus group discussions (FGD) (see Table 1).

Table 1. Objectives and Methods

Objectives	Methods		
	Delphi Activity with International Experts	*Theory of Change Workshops in Brazil, Nepal, and Nigeria*	*Qualitative Interviews in Brazil, Nepal, Nigeria, and the UK*
Objective 1: Cultural concepts of depression, depression risk, and health systems	Experts will comment on risk factors in different cultural settings	Policymakers, service providers, parents, adolescents, and other stakeholders will develop a theory of change for depression identification and prevention	Policymakers, service providers, parents, adolescents, and other stakeholders will describe cultural explanatory models of adolescent depression, including help-seeking experiences
Objective 2: Feasibility, acceptability, and perceived utility of a risk calculator for adolescent depression	Experts will comment on feasibility of risk assessment and early detection methods	Policymakers, service providers, parents, adolescents, and other stakeholders will comment on how to determine depression risk in the theory of change model	Policymakers, service providers, parents, adolescents, and other stakeholders will review and comment on a mock-up of a risk calculator
Objective 3: Feasibility and acceptability of biological psychiatry research in low- and middle-income countries	Experts will comment on specificity and feasibility of risk factors in different cultural contexts	Policymakers, service providers, parents, adolescents, and other stakeholders will comment on the role of biological research within the theory of change model	Policymakers, service providers, parents, adolescents, and other stakeholders will describe priorities, barriers, and utility of biological research in their settings

Delphi activity:

A Delphi panel consensus study will be conducted soliciting opinions from global experts in the field of adolescent depression. The Delphi method allows for the systematic generation and scoring of research questions using predetermined criteria, and has been widely used for consensus studies in depression research.[14-19] The Delphi panel will provide a state of the field recommendation on the range and relevance of biopsychosocial risk factors for adolescent depression, strategies for early identification of depression in adolescence, and the feasibility for research and preventive interventions, in heterogeneous global settings.

The results of a systematic literature review of common biopsychosocial risk factors, a separate component of the IDEA research portfolio, will inform the development of the Delphi questionnaires.[20] Three rounds of surveys will be administered in the Delphi activity.

For peer review only - http://bmjopen.bmj.com/site/about/guidelines.xhtml

Respondents will generate a list of biopsychosocial risk factors, possible early signs, and early detection strategies for adolescent depression in Round-1 via an open-ended free listing exercise. The results will be collated via categorization of similar items into a final list. The finalized items from Round-1 will be ranked by panelists according to feasibility and specificity in Round-2. From the ranking exercise, measures of centrality will be generated, including frequency, average rank, and Smith's salience index, for each item.[21] In Round-3, panelists will be provided with these group summary statistics, and given the opportunity to compare and change their rankings, to derive expert consensus. Respondents will provide in-depth commentary on how ranks can vary according to cultural factors in Round-3 as well. The web-based survey software, Qualtrics, will be used to implement the Delphi activity.[22]

Theory of Change Workshops:

Theory of Change (ToC) methodology has emerged as a viable alternative to conceptualizing program design and evaluation in global mental health.[23] The ToC method offers a theory-driven approach which identifies and lays out causal pathways that lead to the outcome of a program or process. A ToC explicitly includes the short-, medium-, and long-term outcomes that lead up to an expected impact, the interventions and indicators that constitute the program and measure its progress, and relevant programmatic and contextual assumptions.[24]

For the IDEA project, a ToC approach is well-suited to address each of the objectives as it allows country-specific stakeholder groups to conceptualize and create causal pathways of the feasibility and acceptability of conducting biological and psychosocial research and integrating risk and preventative interventions into existing health, education and social services systems of the study sites. Stakeholder driven discussion during ToC workshops facilitated by IDEA researchers will produce visual maps of these pathways in each research site. Soliciting perspectives from diverse stakeholder groups from the socio-ecological hierarchies of these countries will enable the formulation of integrative, country-specific and global theories of change of these causal processes.

Key-informant interviews (KII) & Focus-Group Discussions (FGD):

In order to achieve the three objectives, we will conduct qualitative KIIs and FGDs at each site. Key-informants are individuals with "great knowledge…who can shed light on the inquiry issues" and in-depth semi-structured interviews with key informants will drive the major focus of data collection for this study.[25] Focus group discussions are highly useful in gleaning both commonly shared and divergent views of a group.[26] FGDs are a useful method for collecting insights from relatively homogenous groups or among those who have a shared common experience i.e. lived experience of depression, etc.[25]

The thematic areas that will be explored in the interviews include the pathogenesis and lived experience of depression and the contextual considerations of depression identification mechanisms. This includes the structural and social mediators and moderators of depression manifestation and prevention. We will also explore the pathways of how stakeholders seek help for depression, and the availability, accessibility, and acceptability of treatment modalities. Finally, we will elicit perspectives exploring the pathways that lead towards or prevent successful recovery from depression.

For peer review only - http://bmjopen.bmj.com/site/about/guidelines.xhtml

STUDY SETTINGS: COUNTRY DESCRIPTIONS (1481 words)

Brazil

Brazil is an upper middle-income country representing the growing number of countries with rapidly developing economies and urbanization, collectively known as the BRICS nations (Brazil, Russia, India, China, and South Africa). Two out of every three adolescents worldwide live in a BRICS country. Brazil has a total population of 210 million people; and the prevalence of major depressive disorder has been estimated to be around 9.4 percent among adults, being one of the highest rates in Latin America.[27] The health and education systems are characterized by a mix of public and private services. Overall, the education system is divided into preschool, fundamental school (nine years) and high school (three additional years). The public Unified Health System provides universal health coverage free of charge. Mental health care is provided at multiple levels of complexity, including psychosocial centers (2,462 units in 2017, with only 229 specialized to the care of children and adolescents).[28] The social services system is organized based on the specificities of each territory. The child care council is the organ responsible for ensuring the rights of children and adolescents. [29]

In Brazil, the qualitative study will be implemented by a team from the Universidade Federal do Rio Grande do Sul. Data collection efforts will be led by five researchers from IDEA Brazil team: one psychologist, two psychology undergraduate students, and one with social communication training (all females); and two medical doctors (both males), which includes an IDEA principal investigator. The recruitment process will utilize connections of the IDEA Brazil team to local, relevant, institutions. The KIIs will take place in private and convenient locations for each participant, and the FGDs will be held in Hospital de Clínicas de Porto Alegre.

Nepal

Nepal is one of the poorest countries in the world and represents conditions of adolescents living in the least-developed nations. Nepal also is representative of the large number of adolescents living in humanitarian settings. Adolescents in Nepal today grew up during a decade-long civil war, and many have been impacted by repeated environmental disasters including earthquakes, flooding, and landslides. More than 20 percent of the national population have at least one mental health problem in Nepal.[30] Adolescents comprise 24 percent of the total population of Nepal, and 14 percent of this population have one or more psychosocial problems.[31, 32] Known risk factors contributing to depression include political conflict, caste-based disparities, earthquake, urban habitation, domestic violence, poverty, and other social factors.[33-35] There is a single outpatient child and adolescent psychiatry clinic and no corresponding inpatient facilities. The National Mental Health Policy was developed in Nepal in 1996 to integrate mental health into primary health care services.[36] The Ministry of Health and Population has adapted the World Health Organization's (WHO) Mental Health Gap Action Programme (mhGAP), developed a community mental health care package training curricula to non-specialized mental health service providers, revised psychotropic medications in the essential drug list, included four priority Mental Neurological and Substance use disorders (depression, psychosis, epilepsy and alcohol use disorder) in basic health service package and revised the National Adolescent Health and Development Strategy.[37] The Ministry of Health & Population has recently been drafting National Mental Health Strategy and

For peer review only - http://bmjopen.bmj.com/site/about/guidelines.xhtml

Action Plan in an attempt to reinforce integration of mental health into primary care with a focused strategy on child and adolescent mental health which was not included in the National Mental Health Policy 1996. The Ministry of Education has recently started piloting the introduction of school nurses as an initiative to improve child and adolescent health in selected regions of the country.

The data collection efforts in Nepal will be implemented by the Transcultural Psychosocial Organization Nepal (TPO Nepal), one of Nepal's leading psychosocial organizations for providing clinical care and conducting mental health research. TPO interviewers and FGD facilitators will include 2 female researchers with graduate level training in Public Health and 1 male researcher with graduate training in Management Studies, under the guidance of a senior TPO director and researcher, who is an IDEA study Co-investigator. Recruitment for the study will be facilitated by reaching out to institutions offering adolescent mental health services in Kathmandu district in Nepal. KIIs will be conducted in private locations at health facilities, schools, stakeholder offices, TPO Nepal offices, Ministry offices, etc. The FGDs will each be conducted at TPO offices. KIIs and FGDs will be primarily conducted in Nepali & English.

Nigeria

Nigeria is a lower-income country and the most populous country in Africa. The experiences of adolescents in Nigeria reflect the rapid development of African economies as well as chronic exposure to political violence, community violence, and high rates of infectious diseases, including HIV/AIDS. In Nigeria, adolescents (age 10-19 years) make up approximately 41 million, or 23 percent of the total population of 180 million.[38] In 2015, the WHO estimated the prevalence of depression to be approximately 3.9 percent in the general population.[39] However, nationally representative research has indicated that up to 10 percent of the Nigerian school adolescents to have clinical depression.[40] With over 15 million adolescents in its secondary schools, Nigeria has an estimated 1.5 million depressed school adolescents in need of intervention.[41] Mental health remains under-prioritized, and is unaddressed in the National Health Policy and National Strategic Health Development Plan (2010-2015), as well as in educational and criminal justice policies.[42] Similarly, child and adolescent mental health are not mentioned in the National Child Health Policy. Additionally, the School Health Policy launched by the Federal Government in 2006 did not have provisions for mental health.[43] There are no government-run health facilities specifically for children and adolescents, and less than 20 percent of primary and secondary schools have activities that promote mental health.[44] Social and health risk factors for poor mental health, particularly for children and adolescents, include high levels of poverty and child malnutrition, as well as the prevalence of child labor and under-age marriage.[45] In Lagos State, where the IDEA project will be implemented, there are over 1 million adolescents in its nearly 3,000 secondary schools.[46]

In Nigeria, the study is managed by the Lagos State University, College of Medicine, Department of Behavioral Medicine. Data collection efforts will be led by a team of 2 female doctors, a Consultant Psychiatrist and a Medical Officer under guidance of a senior Professor, who is an IDEA project co-investigator. Recruitment will be through flyers distributed to social workers, health care providers and the teachers in both public and private schools in Lagos. Letters will be written to the permanent secretaries of the ministries of health, education, social services and the health service commission requesting the nomination of civil servants

For peer review only - http://bmjopen.bmj.com/site/about/guidelines.xhtml

for policy-maker interviews of the respective ministries. Interviews will be done at the convenience of the participant, and the timing and the location will be flexible. Interviews can be held at the office of the researchers or at the offices of the person being interviewed. The FGDs will be held at department of Behavioural Medicine of The Lagos State University Teaching Hospital and at the Child and Adolescent Mental Health Service of The Federal Neuropsychiatry Hospital Oshodi Annex.

United Kingdom

The UK is the study setting representative of adolescents living in highly resourced regions such as Western Europe and North America. Adolescents aged 10-19 years old comprise approximately 12 percent of the total population of the UK, of whom 17 percent (2.3 million children) are living in persistent poverty, and 18 percent do not attain a baseline level of proficiency in reading to enable them to participate effectively in activities requiring literacy.[47-49] In the UK, one in seven 11-16 year-olds has at least one mental illness, however, only 25 percent of these receive support from the National Health Service (NHS).[50] The NHS Long Term Plan[51] highlights prevention as a national priority for mental health. Members of the UK also have their own mental health policies, strategies, or programs for the general population. England, Scotland and Wales have national mental health strategies and policies that support those with mental illnesses by promoting anti-stigma campaigns and recommendations for health providers. In the UK, Child and Adolescent Mental Health Services (CAMHS) are provided by the NHS and cover community and hospital-based care up to 17 years of age. Countries within the UK each feature some variant of a task force or program responsible for improving the quality and accessibility of health care for children and young people, aiming to increase government funding, decrease waiting times, improve school counseling services, and increase social services provision to support child development.[50, 52]

In the UK, the data collection efforts are implemented by King's College London. The primary data collector is a female social scientist with graduate training in nursing and psychology, with experience in conducting qualitative research. The UK activities are guided by a co-investigator of the IDEA project. Participants will be recruited using an opportunistic sampling method, with stakeholder specific adverts being circulated via professional networks, social media, and placed in key locations in the community, including local NHS hospitals. The KIIs will take place at either one of the King's College London campuses, stakeholder's office spaces where appropriate, or over the telephone.

STUDY PARTICIPANTS

Delphi Activity

A small international steering committee will be formed to guide the Delphi activity. To identify panelists, key publications in the field will be reviewed and authors will be invited to participate via email. Additional panelists will be identified and recommended by the Delphi steering committee. Respondent profiles will include researchers and academics, clinicians and service providers, and policymakers from diverse global and economic settings. The sample required for reliability in consensus methodology is between 6 and 15 participants, and this study will aim to establish a panel of 20 global experts.[53]

For peer review only - http://bmjopen.bmj.com/site/about/guidelines.xhtml

Theory of Change

The ToC workshops will be comprised of approximately 6-12 participants and vary according to context-specific needs. In Brazil, we will conduct 4 ToC workshops with researchers, adolescents, parents, and policymakers. We will conduct 3 ToC workshops in Nepal: one with adolescents and youth researchers; one with parents/guardians, schoolteachers and service providers (clinical psychologists); and one with clinicians and policymakers. In Nigeria, we will conduct 2 ToC workshops with adolescents, parents, NGO leaders, health service providers, teachers, and policymakers. ToC workshops will not be conducted in the UK because a lot of research on the neurobiological underpinnings of adolescent mental health is already occurring in this context. Rather, the UK team will conduct interviews with global health academics to gain better understandings regarding their views on the barriers and facilitators to conducting such research in LMIC contexts.

Key Informant Interviews and Focus Group Discussions

For this study, we seek to generate a sample consisting of diverse stakeholders that can provide unique perspectives and help constitute an integrated understanding of the phenomena and processes under investigation. We will engage primary stakeholders such as adolescents (those with both a history of depression and those without) and their parents. Secondary stakeholders will be recruited from the socio-ecological systems and institutions that develop and nurture the environments which shape the experiences of the primary stakeholders. These include schoolteachers and counselors, health service providers, social workers and policymakers. Accordingly, we use a purposeful sampling strategy to solicit key-informants with direct experience and deep insight of the inquiry topics (e.g. adolescents and parents), or due to the strategic positions they occupy in the health and education systems and social services (e.g. social workers or policymakers).[25]

This multi-perspective approach will allow triangulation of the data through comparison of insights between, and across, stakeholder groups. For KIIs, a sample of 12 qualitative interviews is sufficient for identification of themes in relatively homogeneous groups.[54] Only those policymakers who hold relevant roles and expertise will be recruited for KIIs. Each FGD will be comprised of approximately 8-12 participants. In each site the sample will vary according to context-specific priorities. Table 2 presents the various stakeholder groups targeted for KIIs and FGDs.

Table 2: Key informant interviews & Focus group discussion stakeholders in the IDEA Project

Stakeholders	Brazil		Nepal		Nigeria		UK*
	KII	FGD	KII	FGD	KII	FGD	KII
Adolescents	6	1	12			1	
Health service providers	12		12		12		12
Policymakers	6		6		6	1	12
Parents	6	1	6	2		1	12
Researchers & academics							12
School teachers & counselors	12		12		12		12

For peer review only - http://bmjopen.bmj.com/site/about/guidelines.xhtml

Social workers	12		12		12		12

*No FGDs to be conducted in the UK

For each stakeholder group indicated in Table 2, relevant inclusion and exclusion criteria apply. Health service providers need to have training and experience working with adolescents and/or mental health. These can include psychiatrists, psychologists, pediatricians, and general physicians. Similarly, we will include those social workers who have experience in working with adolescents. For educators, we will only include teachers and school counselors who work with adolescent students and exclude those working with younger children in the school system. Researchers and academics will be recruited based on expertise in adolescent depression. Policymakers will be included based on relevant linkages to the health, education, and social care systems, and the ability to comment meaningfully on the programmatic and policy environments of the country. Finally, to get rich narratives on context-specific lived experience of depression we will recruit adolescents with current or past history of depression. We will also interview adolescents with no history of depression to broaden our understanding of the knowledge and attitudes surrounding depression. Parents of these two subsets of adolescents will be recruited to gain perspectives of parental attitudes towards, and knowledge of depression, and understand its impact on families.

DATA COLLECTION (KII and FGD)

The KII and FGD guides will be developed using an iterative process. We will first create a deductive, theory-informed interview guide and conduct approximately 6 preliminary KIIs with different stakeholders, across each site. The data from these initial KIIs will be used to revise the guides for contextual sensitivity. The subsequent KIIs and FGDs will be conducted with these revised guides, with the provision for further edits if subsequent findings indicate it to be necessary. Please refer to Figure 1 for an overview of the data collection and data analysis process.

All data collection procedures and decisions will be collated into memos to preserve an auditable record of methodological decisions. KIIs and FGDs will be audio recorded with consent (and assent where applicable), and audio recordings will be professionally translated to English (except brazil, where analysis will be conducted in Portuguese) for data analysis. Local terminology for depression and idioms of distress will be included in the local language along with English translations.

Researchers will complete debriefing forms to capture salient ideas, important exchanges, salient events, and other features of the interaction during KIIs and FGDs. Debriefing forms are an essential component of the data analysis process and are intended to provide meaning and understanding of the culture, social situation, or phenomenon under investigation, and contextualize the interview transcript.[25, 55]

DATA ANALYSIS (KII and FGD)

The major deductive themes of the qualitative study are as follows:

- Understanding of developmental, social, and health changes in adolescents

For peer review only - http://bmjopen.bmj.com/site/about/guidelines.xhtml

- Understanding of depression in adolescents: symptoms, impact, and help-seeking (e.g., how can depression be detected early and where, whom, and when would be best to identify it early)
- Perception of causes or contributory factors to depression in adolescents
- Views regarding possible preventive measures (primary and secondary prevention), including their feasibility, acceptability, and utility

We will use framework analysis (FA) to guide the data collection and analysis of this study[56, 57] following the stages of the FA approach in applied qualitative research:

1. Transcription
2. Familiarization with the interview
3. Coding
4. Developing a working analytical framework
5. Applying the analytical framework
6. Charting data into the framework matrix
7. Interpreting the data

We will adopt a modified approach to implementing the framework approach, as presented in Figure 1. The first step will be to create the KII and FGD interview guides and develop a universal codebook that can be used across the four countries. This deductive codebook will be derived from the study objectives, existing literature, theory, and expert knowledge. Each country will adjust the codebook to include country-specific codes as necessary. The methodological approach to data collection and analysis will be cyclical and iterative, with each step informing and enhancing subsequent steps, as presented in Figure 1.

<insert Figure-1 here>

Figure 1: Data collection and analysis approach.

IDEA researchers across the four country sites will code the data. Codes and themes will be refined using the constant comparison method.[58] This involves moving back and forth between newly coded data and comparing it with previously coded segments to check that the code is still capturing the same essence in the excerpts, as data analysis continues to mature. The addition of new codes and themes will continue until no new codes, categories, or themes emerge from the data, indicating theoretical saturation.[59]

Throughout analysis, researchers will maintain memos to capture ideas, themes, problems, etc., that are extracted from the coding process. Inductive themes can be identified at any stage during the analysis. We will engage in a reflexive approach to data analysis, critically reflecting upon the theoretical structures that are drawn out of the analysis.[60] Wherever applicable we will reconceptualize the evidence using other possible theoretical and conceptual models, to test, validate or refine ideas and findings.

Once coding is completed, a summary of each code will be written capturing the essence of that code across the whole dataset, with supporting quotes and researcher insights.

For peer review only - http://bmjopen.bmj.com/site/about/guidelines.xhtml

We will stratify results according to site and stakeholder characteristics to gain nuanced understandings of how themes vary and converge across groups. In a final step, thematic narratives will be developed in preparation for article manuscripts. We will use NVivo version-12 software for coding and analysis.[61]

ETHICS AND DISSEMINATION

The IDEA qualitative study has been reviewed and approved by the institutional review board of the George Washington University, USA. Additionally, the study has country-specific approvals from the Nepal Health Research Council in Nepal; the Ethics Committee at Hospital de Clínicas de Porto Alegre, in Brazil; the Lagos State University Teaching Hospital Research and Ethics Committee and The Research and Ethics Committee of The Federal Neuropsychiatry Hospital Yaba, Lagos, in Nigeria; and the Psychiatry, Nursing and Midwifery Research Ethics Subcommittee at King's College London, in the UK.

All data collection will operate under the ethical principles of informed consent and assent. Data from the project will be de-identified and stored in password-protected computer servers with access restricted to essential study personnel only. All results will be presented in the aggregate to minimize any potential risks to confidentiality of research participants. Study findings will be reported via publications in academic journals and conferences. Results will also be disseminated through traditional and social media, wherever applicable. We will follow the Consolidated criteria for Reporting Qualitative research (COREQ) guidelines in resultant publications.[62]

DISCUSSION

In this protocol we discuss a systematic and progressive set of methods to understand the complex problem of contextual variations in identifying depression early in adolescence globally. Through this research we seek to establish the acceptability, feasibility and utility of integrating such approaches in Brazil, Nepal, Nigeria, and the UK. Approaches determined to be culturally acceptable, feasible, and effective could then be proposed for health providers to support the early detection of depression or identification of risk factors. Identifying depression early in adolescence and understanding the risk and protective factors of depression would inform policy decisions for funding allocation, amendments to legislation, and programming for interventions. Although the prominence of mental health services in health systems varies across countries of different income classifications, compiling stakeholder perspectives can help tailor national mental health policies toward the needs of the specific country. For instance, revelations regarding the effectiveness of existing policies or the level of public awareness about mental illness provide national and community leadership with the context necessary to actualize improvements in current mental health infrastructure.

Investigating these issues using qualitative methods in multiple global settings has not been conducted to date. Therefore, this study will contribute to addressing this gap in the literature. Additionally, there is growing movement and discussion on the transparency, reliability, validity and reproducibility of qualitative research.[63] A systematic presentation of the conceptualization and procedural implementation of such studies can contribute to the discussion surrounding the transparency and veracity of qualitative methods, and its utility for global mental health research.

For peer review only - http://bmjopen.bmj.com/site/about/guidelines.xhtml

The variety of stakeholders to be interviewed presents the opportunity for a range of solutions to be developed across sectors, potentially in the form of public campaigns, health governance restructuring, or additions to school resources. Engaging community members and leaders in discussions surrounding adolescent depression is crucial for the sustainability of these changes. By investigating these issues across a range of low-, middle- and high-income settings, we hope to generate context-specific, and potentially global understandings of, and responses to, depression among the world's 1.2 billion adolescents.

REFERENCES

1. Belfer, M.L., *Child and adolescent mental disorders: the magnitude of the problem across the globe.* J Child Psychol Psychiatry, 2008. **49**(3): p. 226-36.
2. Thapar, A., et al., *Depression in adolescence.* Lancet, 2012. **379**(9820): p. 1056-67.
3. Avenevoli, S., et al., *Major depression in the national comorbidity survey-adolescent supplement: prevalence, correlates, and treatment.* Journal of the American Academy of Child and Adolescent Psychiatry, 2015. **54**(1): p. 37-44.e2.
4. Cipriani, A., et al., *Comparative efficacy and tolerability of antidepressants for major depressive disorder in children and adolescents: a network meta-analysis.* The Lancet, 2016. **388**(10047): p. 881-890.
5. Kieling, C., et al., *Identifying depression early in adolescence.* The Lancet Child & Adolescent Health, 2019. **3**(4): p. 211-213.
6. Henrich, J., S.J. Heine, and A. Norenzayan, *The weirdest people in the world?* Behav Brain Sci, 2010. **33**(2-3): p. 61-83; discussion 83-135.
7. Ormel, J., et al., *Prevention of depression will only succeed when it is structurally embedded and targets big determinants.* World Psychiatry, 2019. **18**(1): p. 111-112.
8. Lindström, J. and J. Tuomilehto, *The Diabetes Risk Score.* Diabetes Care, 2003. **26**(3): p. 725.
9. Lloyd-Jones, D.M., *Cardiovascular risk prediction: basic concepts, current status, and future directions.* Circulation, 2010. **121**(15): p. 1768-77.
10. Parker, J.S., et al., *Supervised risk predictor of breast cancer based on intrinsic subtypes.* Journal of clinical oncology : official journal of the American Society of Clinical Oncology, 2009. **27**(8): p. 1160-1167.
11. Gautam, K., et al., *Ethical considerations for conducting cross-cultural biological psychiatry and prevention research on depression among adolescents in low- and middle-income countries*, in *Developments in Neuroethics and Bioethics*. 2019, Academic Press.
12. Cuijpers, P., A.T. Beekman, and C.F. Reynolds, 3rd, *Preventing depression: a global priority.* Jama, 2012. **307**(10): p. 1033-4.
13. Brent, D.A., et al., *Effect of a Cognitive-Behavioral Prevention Program on Depression 6 Years After Implementation Among At-Risk Adolescents: A Randomized Clinical Trial.* JAMA Psychiatry, 2015. **72**(11): p. 1110-8.
14. Cairns, K.E., et al., *Identifying prevention strategies for adolescents to reduce their risk of depression: A Delphi consensus study.* J Affect Disord, 2015. **183**: p. 229-38.
15. Morgan, A.J. and A.F. Jorm, *Self-help strategies that are helpful for sub-threshold depression: a Delphi consensus study.* J Affect Disord, 2009. **115**(1-2): p. 196-200.

For peer review only - http://bmjopen.bmj.com/site/about/guidelines.xhtml

16. Langlands, R.L., et al., *First aid for depression: a Delphi consensus study with consumers, carers and clinicians.* J Affect Disord, 2008. **105**(1-3): p. 157-65.
17. Yap, M.B., et al., *Parenting strategies for reducing the risk of adolescent depression and anxiety disorders: a Delphi consensus study.* J Affect Disord, 2014. **156**: p. 67-75.
18. Grunder, G., et al., *[Rank of outcome parameters in the treatment of depression : Results of a Delphi panel survey].* Nervenarzt, 2017. **88**(9): p. 1044-1049.
19. Jones, J. and D. Hunter, *Consensus methods for medical and health services research.* Bmj, 1995. **311**(7001): p. 376-80.
20. Pedersen, G.A., et al., *Protocol for a systematic review of the development of depression among adolescents and young adults: psychological, biological, and contextual perspectives around the world.* Systematic Reviews, 2019. **8**(1): p. 179.
21. Smith, J.J. and S.P. Borgatti, *Salience Counts—And So Does Accuracy: Correcting and Updating a Measure for Free-List-Item Salience.* Journal of Linguistic Anthropology, 1997. **7**(2): p. 208-209.
22. Qualtrics, *Qualtrics*. 2019, Qualtrics: Provo, Utah, USA.
23. De Silva, M.J., et al., *Theory of Change: a theory-driven approach to enhance the Medical Research Council's framework for complex interventions.* Trials, 2014. **15**: p. 267.
24. Vogel, I., *Review of the Use of 'Theory of Change' in International Development*. 2012, UK Department of International Development.
25. Patton, M.Q., *Qualitative Research & Evaluation Methods: Integrating Theory and Practice*. 4th ed. 2014, Thousand Oaks, CA: Sage Publications.
26. Remler, D.K. and G.G. Van Ryzin, *Research Methods in Practice*. 2015, Los Angeles | London | New Delhi: SAGE.
27. Andrade, L.H., et al., *Mental disorders in megacities: findings from the Sao Paulo megacity mental health survey, Brazil.* PLoS One, 2012. **7**(2): p. e31879.
28. Trape, T.L. and R.O. Campos, *The mental health care model in Brazil: analyses of the funding, governance processes, and mechanisms of assessment.* Rev Saude Publica, 2017. **51**(0): p. 19.
29. Ministry of Social Development and Fight Against Hunger. 2011.
30. Risal, A., et al., *Anxiety and depression in Nepal: prevalence, comorbidity and associations.* BMC Psychiatry, 2016. **16**(1): p. 102.
31. Adhikari R.P., et al., *Health and Social Vulnerability of Adolescents in Nepal.* SM Journal of Public Health and Epidemiology, 2016. **2**(3): p. 1032.
32. Central Bureau of Statistics, *National Population and Housing Census 2011,*. 2011, Government of Nepal: Kathmandu, Nepal.
33. Upadhaya, N., et al., *Geographies of adolescent distress: A need for a community-based psychosocial care system in Nepal.* Intervention, 2018. **17**.
34. Kohrt, B.A., et al., *Political violence and mental health in Nepal: prospective study.* Br J Psychiatry, 2012. **201**(4): p. 268-75.
35. Kohrt, B.A., et al., *Social ecology of child soldiers: child, family, and community determinants of mental health, psychosocial well-being, and reintegration in Nepal.* Transcultural psychiatry, 2010. **47**(5): p. 727-753.
36. Luitel, N.P., et al., *Mental health care in Nepal: current situation and challenges for development of a district mental health care plan.* Conflict and health, 2015. **9**: p. 3-3.

For peer review only

For peer review only - http://bmjopen.bmj.com/site/about/guidelines.xhtml

37. Kohrt, B.A., et al., *How competent are non-specialists trained to integrate mental health services in primary care? Global health perspectives from Uganda, Liberia, and Nepal.* Int Rev Psychiatry, 2018. **30**(6): p. 182-198.
38. Affairs, U.N.D.o.E.a.S., *World Population Prospects* 2019.
39. World Health Organization, *Depression and other common mental disorders: Global health estimates*. 2017, World Health Organization: Geneva.
40. Adewuya, A.O., B.A. Ola, and O.O. Aloba, *Prevalence of major depressive disorders and a validation of the Beck Depression Inventory among Nigerian adolescents.* Eur Child Adolesc Psychiatry, 2007. **16**(5): p. 287-92.
41. National Population Commission (NPC) [Nigeria] and ICF International, *Nigeria Demographic and Health Survey 2013*. 2014, NPC and ICF International: Abuja, Nigeria, and Rockville, maryland.
42. Abdulmalik, J., L. Kola, and O. Gureje, *Mental health system governance in Nigeria: challenges, opportunities and strategies for improvement.* Global mental health (Cambridge, England), 2016. **3**: p. e9-e9.
43. Federal Ministry of Education Nigeria, *National School Health Policy*. 2006: Abuja, Nigeria.
44. World health Organization, M.o.H.N., *WHO-AIMS report on Mental Health System in Nigeria,*. 2006: Ibadan, Nigeria.
45. Atilola, O., et al., *State of the Nigerian child - neglect of child and adolescent mental health: a review.* Paediatr Int Child Health, 2015. **35**(2): p. 135-43.
46. Education Sector Support Programme in Nigeria (ESSPIN), *Lagos Private School Census 2010-2011 Report*. 2011, ESSPIN: Lagos, Nigeria.
47. Hagell, A., J. Coleman, and F. Brooks, *Key Data on Adolescence 2015*. 2015, Association for Young People's Health: London.
48. Social Metrics Commission, *Measuring poverty 2019*. 2019: United Kingdom.
49. Organization for economic cooperation and development, *United Kingdom Country Note – Results from PISA 2015*. 2019.
50. National Health Service, *Mental Health of Children and Young People in England*. 2018.
51. National Health Service, *The NHS Long Term Plan*. 2019.
52. Health and Social Care, *Children and Young People's Mental Health Taskforce: delivery plan*. 2018, Government of Scotland.
53. Murphy, M.K., et al., *Consensus development methods, and their use in clinical guideline development.* Health Technol Assess, 1998. **2**(3): p. i-iv, 1-88.
54. Guest, G., A. Bunce, and L. Johnson, *How Many Interviews Are Enough?* Field Methods, 2006. **18**(1): p. 59-82.
55. Emerson, R., R. Fretz, and L. Shaw, *Writing ethnographic fieldnotes*. 1995, Chicago: University of Chicago Press.
56. Gale, N.K., et al., *Using the framework method for the analysis of qualitative data in multi-disciplinary health research.* BMC Medical Research Methodology, 2013. **13**(1): p. 117.
57. Smith, J. and J. Firth, *Qualitative data analysis: the framework approach.* Nurse Res, 2011. **18**(2): p. 52-62.

For peer review only - http://bmjopen.bmj.com/site/about/guidelines.xhtml

58. Glaser, B.G. and A.L. Strauss, *The Discovery of Grounded Theory: Strategies for Qualitative Research*. 1967, Chicago: Aldine.
59. Strauss, A. and J. Corbin, *Basics of Qualitative Research: Grounded Theory procedures and techniques*. 1990, Newbury Park, CA: SAGE.
60. Finlay, L., *Negotiating the swamp: the opportunity and challenge of reflexivity in research practice.* Qualitative Research, 2002. **2**(2): p. 209-230.
61. Richards, L., *Using NVivo in qualitative research*. 1999, London ;: Sage Publications.
62. Tong, A., P. Sainsbury, and J. Craig, *Consolidated criteria for reporting qualitative research (COREQ): a 32-item checklist for interviews and focus groups.* International Journal for Quality in Health Care, 2007. **19**(6): p. 349-357.
63. Tsai, A.C., et al., *Promises and pitfalls of data sharing in qualitative research.* Soc Sci Med, 2016. **169**: p. 191-198.

For peer review only

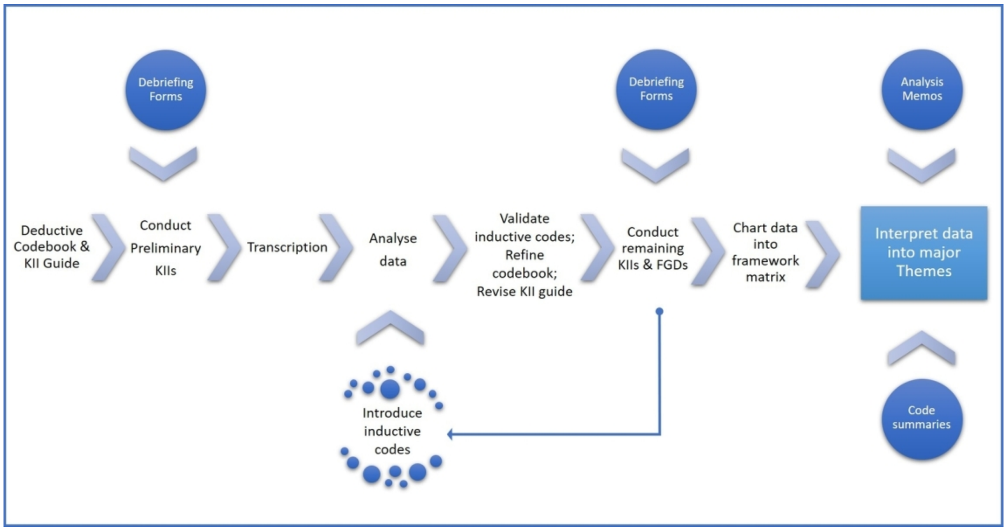

Figure 1: Data collection and analysis approach.

138x72mm (300 x 300 DPI)

BMJ Open

Detection of risk for depression among adolescents in diverse global settings: Protocol for the IDEA qualitative study in Brazil, Nepal, Nigeria and the United Kingdom

Journal:	*BMJ Open*
Manuscript ID	bmjopen-2019-034335.R1
Article Type:	Protocol
Date Submitted by the Author:	12-May-2020

Complete List of Authors:	Wahid, Syed; George Washington University, Division of Global Mental Health<br>Pedersen, Gloria; George Washington University,<br>Ottman, Katherine; George Washington University, Division of Global Mental Health<br>Burgess, Abigail; King's College London, Psychology and Neuroscience<br>Gautam, Kamal; Transcultural Psychosocial Organization Nepal<br>Martini, Thais; Universidade Federal do Rio Grande do Sul, Department of Psychiatry<br>Viduani, Anna; Universidade Federal do Rio Grande do Sul, Department of Psychiatry<br>Momodu, Olufisayo; Lagos Island General Hospital, Department of Psychiatry<br>Lam, Crystal; George Washington University, Department of Global Health<br>Fisher , Helen; King's College London, SGDP Centre<br>Kieling, Christian; Universidade Federal do Rio Grande do Sul, Department of Psychiatry; Hospital de Clínicas de Porto Alegre, Child & Adolescent Psychiatry Division<br>Adewuya, Abiodun O; Lagos State University College of Medicine,<br>Mondelli, Valeria; King's College London<br>Kohrt, Brandon A.; Duke Univ; George Washington University, Division of Global Mental Health

**Primary Subject Heading**:	Mental health
Secondary Subject Heading:	Global health, Qualitative research
Keywords:	adolescent, depression, risk factors, protective factors, qualitative

SCHOLARONE™
Manuscripts

I, the Submitting Author has the right to grant and does grant on behalf of all authors of the Work (as defined in the below author licence), an exclusive licence and/or a non-exclusive licence for contributions from authors who are: i) UK Crown employees; ii) where BMJ has agreed a CC-BY licence shall apply, and/or iii) in accordance with the terms applicable for US Federal Government officers or employees acting as part of their official duties; on a worldwide, perpetual, irrevocable, royalty-free basis to BMJ Publishing Group Ltd ("BMJ") its licensees and where the relevant Journal is co-owned by BMJ to the co-owners of the Journal, to publish the Work in this journal and any other BMJ products and to exploit all rights, as set out in our licence.

The Submitting Author accepts and understands that any supply made under these terms is made by BMJ to the Submitting Author unless you are acting as an employee on behalf of your employer or a postgraduate student of an affiliated institution which is paying any applicable article publishing charge ("APC") for Open Access articles. Where the Submitting Author wishes to make the Work available on an Open Access basis (and intends to pay the relevant APC), the terms of reuse of such Open Access shall be governed by a Creative Commons licence – details of these licences and which Creative Commons licence will apply to this Work are set out in our licence referred to above.

Other than as permitted in any relevant BMJ Author's Self Archiving Policies, I confirm this Work has not been accepted for publication elsewhere, is not being considered for publication elsewhere and does not duplicate material already published. I confirm all authors consent to publication of this Work and authorise the granting of this licence.

For peer review only

Title: Detection of risk for depression among adolescents in diverse global settings: Protocol for the IDEA qualitative study in Brazil, Nepal, Nigeria, and the United Kingdom

Running Head: Protocol for the IDEA qualitative study on risk for adolescent depression

Key words: adolescent; depression; risk factors, protective factors; qualitative; framework analysis; delphi method; theory of change.

Authors:

Syed Shabab Wahid
Division of Global Mental Health, George Washington University, Washington DC, United States.

Gloria A. Pedersen
Division of Global Mental Health, George Washington University, Washington DC, United States.

Katherine Ottman
Division of Global Mental Health, George Washington University, Washington DC, United States.

Abigail Burgess
Social, Genetic & Developmental Psychiatry Centre, Institute of Psychiatry, Psychology and Neuroscience, King's College London, London, United Kingdom.

Kamal Gautam
Transcultural Psychosocial Organization Nepal (TPO Nepal), Baluwatar, Kathmandu, Nepal

Thais Martini
Department of Psychiatry, Universidade Federal do Rio Grande do Sul, Porto Alegre, RS, Brazil.

Anna Viduani
Department of Psychiatry, Universidade Federal do Rio Grande do Sul, Porto Alegre, RS, Brazil.

Olufisayo Momodu
Department of Psychiatry, Lagos Island General Hospital, Lagos, Nigeria.

Crystal Lam
Department of Global Health, George Washington University, Washington DC, United States.

Helen L. Fisher*
Social, Genetic & Developmental Psychiatry Centre, Institute of Psychiatry, Psychology & Neuroscience, King's College London, London, United Kingdom.

For peer review only - http://bmjopen.bmj.com/site/about/guidelines.xhtml

Christian Keiling
Department of Psychiatry, Universidade Federal do Rio Grande do Sul and Child & Adolescent Psychiatry Division, Hospital de Clínicas de Porto Alegre, Porto Alegre, RS, Brazil.

Abiodun Adewuya
Department of Behavioural Medicine, Lagos State University College of Medicine, Lagos, Nigeria.

Valeria Mondelli
Department of Psychological Medicine, Institute of Psychiatry, Psychology & Neuroscience, King's College London, London, United Kingdom.

Brandon A. Kohrt
Division of Global Mental Health, George Washington University, Washington DC, United States.

***Corresponding author**:
Helen L. Fisher*
Social, Genetic & Developmental Psychiatry Centre, Institute of Psychiatry, Psychology & Neuroscience, King's College London, 16 De Crespigny Park, London, United Kingdom.
helen.2.fisher@kcl.ac.uk Tel: +44(0)20-7848-5430

Author contributions:
BAK conceived the paper and was in charge of overall direction and planning. SSW, GAP, KO, AB, KG, TM, AV, OM, CL wrote the manuscript with input from all the authors. CK, HLF, AA, VM and BAK provided overall technical review, critical revision, and final approval for publication.

Total words: 4,276 words

Funding
MQ Transforming Mental Health Charity, Brighter Futures grant named "Identifying Depression Early in Adolescence", Grant #MQBF/1 IDEA. Additional support was provided by the UK Medical Research Council (MC_PC_MR/R019460/1) and the Academy of Medical Sciences (GCRFNG\100281) under the Global Challenges Research Fund. HLF is supported by a British Academy Mid-Career Fellowship (MD\170005). CK has received support from Brazilian governmental research funding agencies (Conselho Nacional de Desenvolvimento Científico e Tecnológico [CNPq], Coordenação de Aperfeiçoamento de Pessoal de Nível Superior [CAPES], and Fundação de Amparo à Pesquisa do Estado do Rio Grande do Sul [Fapergs]). VM has been supported by the National Institute for Health Research (NIHR) Mental Health Biomedical Research Centre at South London and Maudsley NHS Foundation Trust and King's College London. The views expressed are those of the authors and not necessarily those of the National Health Service, the NIHR, or the Department of Health and Social Care. BAK is supported by the US National Institute of Mental Health (K01MH104310, R21MH111280).

For peer review only - http://bmjopen.bmj.com/site/about/guidelines.xhtml

Study Dates

The operational dates of the IDEA study are from November 2018 to November 2021.

Competing interests

We declare that we have no competing interests.

Acknowledgement:

We thank the members of the youth advisory boards in the four IDEA country study sites.

For peer review only

For peer review only - http://bmjopen.bmj.com/site/about/guidelines.xhtml

ABSTRACT

Introduction

Globally, depression is a leading cause of disability among adolescents, and suicide rates are increasing among youth. Treatment alone is insufficient to address the issue. Early identification and prevention efforts are necessary to reduce morbidity and mortality. The Identifying Depression Early in Adolescence (IDEA) consortium is developing risk detection strategies that incorporate biological, psychological, and social factors that can be evaluated in diverse global populations. In addition to epidemiological and neuroscience research, the IDEA consortium is conducting a qualitative study to explore three domains of inquiry: (1) cultural heterogeneity of biopsychosocial risk factors and lived experience of adolescent depression in low- and middle-income countries (LMIC); (2) the feasibility, acceptability, and ethics of a risk calculator tool for adolescent depression that can be used in LMIC and high-income countries; and (3) capacity for biological research into biomarkers for depression risk among adolescents in LMIC. This a multi-site qualitative study being conducted in Brazil, Nepal, Nigeria, and the United Kingdom.

Methods and analysis

A systematic set of qualitative methods will be utilized in this study. The Delphi method, Theory of Change (ToC) workshops, key-informant interviews, and focus group discussions will be used to elicit perspectives on the study topics from a broad range of stakeholders (adolescents, parents, policymakers, teachers, health service providers, social workers, and experts). Delphi panelists will participate in 3 survey rounds to generate consensus through facilitated feedback. Stakeholders will create ToC models via facilitated workshops in the LMIC sites. The framework approach will be used to analyze data from the study.

Ethics and dissemination

Ethical approvals were received from the Ethics Review Board of George Washington University and from site-specific institutions in Brazil, Nepal, Nigeria, and the UK. The findings generated from this study will be reported in highly accessed, peer-reviewed, scientific and health policy journals.

ARTICLE SUMMARY

Strengths and limitations of this study

- This study uses the Delphi method, the Theory of Change approach, key-informant interviews and focus group discussion methods in multiple settings varied by culture, economic status, and risk factors to investigate adolescent depression
- The multi-site approach will generate new knowledge on context-specific and universal pathways of adolescent depression manifestation, identification, and prevention
- Perspectives will be elicited from a broad range of stakeholders including adolescents and parents, teachers, health service providers, social workers, and policymakers
- Due to current policy restrictions surrounding research with children, independent adolescent perspectives will not be available from Nigeria. Adolescent interviews were not conducted in the United Kingdom as well.

For peer review only - http://bmjopen.bmj.com/site/about/guidelines.xhtml

- In qualitative focus groups and interviews, individuals with the highest concern may be the most vocal, and thus the results may overrepresent concerns regarding the risk calculator tool

For peer review only

For peer review only - http://bmjopen.bmj.com/site/about/guidelines.xhtml

INTRODUCTION

Globally, depression is a leading cause of illness and disability among adolescents aged 10-19 years old.[1] The presence of depression also increases the risk of suicide, which is the third leading cause of death in this population.[1] As the incidence of depression peaks in adolescence, and often remains undiagnosed, the negative consequences of depression persist as a chronic condition throughout the life course.[2] Major depressive disorder, the most commonly diagnosed form of depression, has a lifetime prevalence of 11 percent representing a major cause of disability across the world.[3]

On a global scale, treatment alone is insufficient to address this problem. Limited efficacy of available interventions along with the limited availability and low-quality of mental health services in many parts of the world persist as significant barriers.[1, 4, 5] Therefore, increased identification of depression early in adolescence and administering preventive strategies become highly salient in addressing this global burden.[5] Understanding the biopsychosocial risk factors that can predict the onset of depression, and the protective factors which can inform measures for preventing its manifestation and severity, are important steps towards achieving this goal.

Research on depression among adolescents in Western Educated Industrialized Rich and Democratic (WEIRD) countries is insufficient to understand the full scale of the problem.[6] Identification and prevention measures need to account for the heterogeneity of risk and protective factors and signs and symptoms of depression, as they manifest across cultures and countries.[7] Therefore, investigating depression across multiple global settings can provide clearer insight into the universal and context-specific risk and protective factors.

Currently, there are three major gaps in global research to address prevention and early identification among adolescents. First, there is a need for context-specific understandings of mental health, adolescent experiences, and risk and protective factors. Cultural conceptions of depression or adolescence vary in their meaning and social implications across populations and context. Accordingly, discerning such factors are crucial in informing culturally sensitive depression identification and prevention efforts.

Second, despite a wide range of research on depression, there is a lack of feasible and acceptable tools to determine the risk of depression among adolescents before the disorder develops. Risk scores are important in other fields of medicine to determine when and how to intervene and how best to allocate resources, e.g., risk determination and treatment provision for cardiovascular disease, diabetes, and cancer.[8-10] Adopting similar tenets to develop a risk calculator for adolescent depression could have substantial public health benefit.

Third, the ethical and institutional policies for research involving adolescents vary widely in low- and middle-income countries (LMIC). Specifically, ethical policies are often lacking for biological psychiatry research, which is needed to shed light on mechanisms and risk markers for adolescent depression.[11] Biological markers may be an important contributor to risk calculation for depression and to evaluate efficacy of prevention efforts.[12] Therefore, assessing the feasibility of implementing biological psychiatry research and determining its ethical and cultural acceptability is critical to identify necessary infrastructural and policy recommendations to increase LMIC capacity for conducting such studies.

To address these three gaps, this article presents the protocol of a global, multi-site, qualitative study which will inform the development of successful adolescent depression

For peer review only - http://bmjopen.bmj.com/site/about/guidelines.xhtml

identification and prevention initiatives. First, in our qualitative study, we will investigate the cultural and contextual perceptions and considerations of identifying adolescent depression, including risk and protective factors, and depression prevention interventions. Second, we will explore the feasibility, acceptability, and utility of a risk calculator for depression in adolescence. Third, we will investigate the feasibility of conducting biological psychiatry research in LMIC settings. To achieve this, a diverse set of qualitative methods will be utilized in four countries: Brazil, Nepal, Nigeria, and the United Kingdom (UK), representing high-, middle- and low-income settings.

OBJECTIVES

Identifying Depression Early in Adolescence (IDEA) Research Consortium

This qualitative study is part of a larger integrated research portfolio being implemented by the Identifying Depression Early in Adolescence (IDEA) research consortium (https://www.mqmentalhealth.org/research/profiles/identifying-depression-early-in-adolescence).[5] The consortium comprises psychiatrists, epidemiologists, neuroscientists, and anthropologists from Brazil, Nepal, Nigeria, the UK, and United States conducting multi-disciplinary research on adolescent depression identification and prevention. The consortium was established with the support of the MQ charity Brighter Futures initiative. The operational dates of the IDEA study are from November 2018 to November 2021.

General Objective of Multi-Component Qualitative Study:

The objective of the current study is to qualitatively explore key topics that will compliment epidemiological and biological psychiatry research being conducted through IDEA. For this multi-component qualitative study, we will consult with adolescents, parents, health care providers, social workers, educators, policymakers and other key stakeholders in Brazil, Nepal, Nigeria and the UK, as well as global experts, about the experience of adolescent depression cross-culturally, the use of a risk calculator for depression during adolescence, and conducting biological psychiatry research with adolescents in LMIC.

Specific Objectives:

1. The qualitative component of IDEA will compare cultural and contextual differences in depression, adolescent experiences, health systems, and health policy across Brazil, Nepal, Nigeria, and the UK. Additionally, information will be collected from an expert panel on risk factors and detection approaches that are specific for adolescent depression and feasible for implementation in LMIC.
2. A risk assessment tool for depression in adolescence has been developed via predictive modeling using cohort data from Brazil, Nigeria, Nepal and the UK.[5, 13, 14] The tool will determine future risk of depression and is derived from research that classifies risk based on the presence of sociodemographic risk factors.[13] This qualitative study will explore the feasibility, acceptability, utility and the ethical implications of such a tool.
3. The IDEA research includes biological psychiatry studies with functional neuroimaging and inflammation pathways. The qualitative work will also explore feasibility, acceptability, and

For peer review only - http://bmjopen.bmj.com/site/about/guidelines.xhtml

ethics related to expanding biological psychiatry research in LMIC and incorporating biological markers to evaluate risk of the onset of depression during adolescence.

STUDY SETTINGS: COUNTRY DESCRIPTIONS

The four IDEA country sites were selected to provide a wide range of contexts to study adolescent depression. Brazil is an upper middle-income country representing the growing number of countries with rapidly developing economies and urbanization, collectively known as the BRICS nations (Brazil, Russia, India, China, and South Africa). Nepal is one of the poorest countries in the world and represents conditions of adolescents living in the least-developed nations. Nepal is also representative of the large number of adolescents living in humanitarian settings due to its recent emergence from a protracted civil war, and frequent environmental disasters. Nigeria is a lower middle-income country and the most populous country in Africa. The experiences of adolescents in Nigeria reflect the rapid development of African economies as well as chronic exposure to political violence, community violence, and high rates of infectious diseases, including HIV/AIDS. The UK is the study setting representative of adolescents living in highly resourced regions such as Western Europe and North America. Further details of the countries and country-specific IDEA research teams are provided in Appendix-1.

METHODS

Conceptual Framework

The IDEA qualitative study is structured according to the social ecological model of health and Singer and Baer's world system theory on the social origins of disease.[15, 16] Using these two guiding theoretical frameworks, we seek to understand the role of individual, interpersonal, institutional, community and policy factors and their interrelations, in depression risk and identification in adolescence (Please refer to Figure-1). Informed by George Engels' classic model, we will elicit biopsychosocial risk and protective factors of depression within and between each ecological stratum.[17] We will utilize Kleinman's Explanatory Model framework to explore the lived experience of depression at the individual level, including culturally driven local idioms of distress.[18, 19] We will further explore how these explanatory models are influenced by relationships at the interpersonal and primary group levels (family and friends), and cultural and social norms at the community level. At the institutional level, we will examine mental health services for depression identification and management, and acceptability and feasibility of risk detection at schools, primary health care, and social services. At the policy level, we will seek to understand challenges and opportunities to facilitate better depression detection and management. Additionally, in the LMIC sites, we will explore institutional capacity for conducting biological psychiatry research (i.e. biological specimen collection, storage, and testing capacity; and research capacity of universities and staff) and policy level considerations for ethical research governance that can support sensitive biological psychiatry research.

<Insert Figure-1>

Figure 1: Conceptual framework for the qualitative component of the IDEA study

For peer review only - http://bmjopen.bmj.com/site/about/guidelines.xhtml

To conduct this study, three methodologies will be used: a Delphi activity, theory of change workshops, and qualitative interviews including both key informant interviews (KII) and focus group discussions (FGD) (see Table 1).

Table 1. Objectives and Methods

Objectives	Methods		
	Delphi Activity with International Experts	*Theory of Change Workshops in Brazil, Nepal, and Nigeria*	*Qualitative Interviews in Brazil, Nepal, Nigeria, and the UK*
Objective 1: Cultural concepts of depression, depression risk, and health systems	Experts will comment on risk factors in different cultural settings	Policymakers, service providers, parents, adolescents, and other stakeholders will develop a theory of change for depression identification and prevention	Policymakers, service providers, parents, adolescents, and other stakeholders will describe cultural explanatory models of adolescent depression, including help-seeking experiences
Objective 2: Feasibility, acceptability, and perceived utility of a risk calculator for adolescent depression	Experts will comment on feasibility of risk assessment and early detection methods	Policymakers, service providers, parents, adolescents, and other stakeholders will comment on how to determine depression risk in the theory of change model	Policymakers, service providers, parents, adolescents, and other stakeholders will review and comment on a mock-up of a risk calculator
Objective 3: Feasibility and acceptability of biological psychiatry research in low- and middle-income countries	Experts will comment on specificity and feasibility of risk factors in different cultural contexts	Policymakers, service providers, parents, adolescents, and other stakeholders will comment on the role of biological research within the theory of change model	Policymakers, service providers, parents, adolescents, and other stakeholders will describe priorities, barriers, and utility of biological research in their settings

Delphi Activity

A Delphi panel consensus study using quantitative and qualitative methods will be conducted soliciting opinions from global experts in the field of adolescent depression. The Delphi method allows for the systematic generation and scoring of research questions using predetermined criteria, and has been widely used for consensus studies in depression research.[20-25] The Delphi panel will provide a state of the field recommendation on the range and relevance of biopsychosocial risk factors for adolescent depression, strategies for early identification of depression in adolescence, and the feasibility for research and preventive interventions, in heterogeneous global settings. Recently, qualitative interviews have been incorporated in Delphi studies to provide meaning to the quantitative results. [26, 27]

Participants: A small international steering committee will be formed to guide the Delphi activity. To identify panelists, key publications in the field will be reviewed and authors will be invited to participate via email. Additional panelists will be identified and recommended by the Delphi steering committee. Respondent profiles will include researchers and academics,

For peer review only - http://bmjopen.bmj.com/site/about/guidelines.xhtml

clinicians and service providers, and policymakers from diverse global and economic settings. The sample required for reliability in consensus methodology is between 6 and 15 participants, and this study will aim to establish a panel of 20 global experts.[28]

Data collection and analysis: The results of a systematic literature review of common biopsychosocial risk factors, a separate component of the IDEA research portfolio, will inform the development of the Delphi questionnaires.[29] Three rounds of surveys will be administered in the Delphi activity. Respondents will generate a list of biopsychosocial risk factors, possible early signs, and early detection strategies for adolescent depression in Round-1 via an open-ended free listing exercise. The results will be collated via categorization of similar items into a final list. The finalized items from Round-1 will be ranked by panelists according to feasibility and specificity in Round-2. From the ranking exercise, measures of centrality will be generated, including frequency, average rank, and Smith's salience index, for each item.[30] In Round-3, panelists will be provided with these group summary statistics, and given the opportunity to compare and change their rankings, to derive expert consensus. The web-based survey software, Qualtrics, will be used to implement rounds 1-3.[31] Following the 3 Delphi rounds, we will conduct in-depth interviews with panelists based on quantitative results. Panelists will provide comments on summary results tables explaining personal and panel rankings, interesting patterns in the quantitative results, and provide narratives on how cultural and contextual factors influence biopsychosocial risk factors. Qualitative data will be analyzed using thematic analysis.[32]

Theory of Change Workshops

Theory of Change (ToC) methodology has emerged as a viable alternative to conceptualizing program design and evaluation in global mental health.[33] The ToC method offers a theory-driven approach which identifies and lays out causal pathways that lead to the outcome of a program or process. A ToC explicitly includes the short-, medium-, and long-term outcomes that lead up to an expected impact, the interventions and indicators that constitute the program and measure its progress, and relevant programmatic and contextual assumptions.[34]

Participants: The ToC workshops will be comprised of approximately 6-12 participants and vary according to context-specific needs. In Brazil, we will conduct four ToC workshops with researchers, adolescents, parents, and policymakers. We will conduct three ToC workshops in Nepal: one with adolescents and youth researchers; one with parents/guardians, schoolteachers, and service providers (clinical psychologists); and one with clinicians and policymakers. In Nigeria, we will conduct two ToC workshops with adolescents, parents, NGO leaders, health service providers, teachers, and policymakers. ToC workshops will not be conducted in the UK because a lot of research on the neurobiological underpinnings of adolescent mental health is already occurring in this context. Rather, the UK team will conduct interviews with global health academics to gain better understandings regarding their views on the barriers and facilitators to conducting such research in LMIC contexts.

Theory of change development: For the IDEA project, a ToC approach is well-suited to address each of the objectives as it allows country-specific stakeholder groups to conceptualize and create causal pathways of the feasibility and acceptability of conducting biological and psychosocial research and integrating risk and preventative interventions into existing health,

For peer review only - http://bmjopen.bmj.com/site/about/guidelines.xhtml

education and social services systems of the study sites. Stakeholder driven discussion during ToC workshops facilitated by IDEA researchers will produce visual maps of these pathways in each research site. Soliciting perspectives from diverse stakeholder groups from the socio-ecological hierarchies of these countries will enable the formulation of integrative, country-specific, and global theories of change of these causal processes.

Key-informant interviews (KII) & Focus-Group Discussions (FGD):

In order to achieve the three objectives, we will conduct qualitative KIIs and FGDs at each site. Key-informants are individuals with “great knowledge…who can shed light on the inquiry issues” and in-depth semi-structured interviews with key informants will drive the major focus of data collection for this study.[35] Focus group discussions are highly useful in gleaning both commonly shared and divergent views of a group.[36] FGDs are a useful method for collecting insights from relatively homogenous groups or among those who have a shared common experience i.e. lived experience of depression, etc.[35]

The thematic areas that will be explored in the interviews include the pathogenesis and lived experience of depression and the contextual considerations of depression identification mechanisms. The experience of depression is heterogenous across the world.[37] Symptoms of anxiety disorder often co-present or can play a role in the onset of depression.[38] Accordingly, in the interviews, we will explore a range of negative affective symptoms by adopting an open-ended approach to probing the signs and symptoms of adolescents’ experience of depression. We will also explore the biopsychosocial risk and protective factors and structural and social mediators and moderators of depression manifestation and prevention. Specifically, we will gather stakeholder perspectives on the cultural acceptability and feasibility of the implementation of a risk calculator for adolescent depression (Please see Box-1).

For peer review only - http://bmjopen.bmj.com/site/about/guidelines.xhtml

The IDEA study risk calculator has been developed as a prognostic model using data from the Pelotas 1993 Birth Cohort, a prospective study conducted in south Brazil.[13] The study included every child born in the city that year: all but 16 mothers agreed to take part, resulting in a total cohort of 5,249 individuals. Follow-up visits were made at multiple time points; the data for the composite risk score development was collected at ages 15 and 18 assessments, which had retention rates 85.7%, and 81.3%, respectively. Using only sociodemographic variables easily obtainable directly from the adolescent at age 15, we developed a risk calculator with good discriminative performance (c-statistic of 0.78) to identify those at high-risk for developing major depression at age 18. A total of eleven variables comprised the final model of the composite score: biological sex, ethnicity, drug use, school failure, social isolation, fight involvement, relationship with mother, relationship with father, relationship between parents, childhood maltreatment, running away from home. As part of IDEA project, the calculator has demonstrated good predictive capacity when externally assessed using data from the Environmental Risk (E-Risk) Longitudinal Twin Study from the United Kingdom, the Dunedin Multidisciplinary and Development Study from New Zealand, and former child soldiers and war-affected adolescents in Nepal.[13, 14] The calculator is being tested among adolescents in Lagos State, in Nigeria as well.

Box 1: IDEA Study risk calculator

We will also explore the pathways of how stakeholders seek help for depression, and the availability, accessibility, and acceptability of treatment modalities. Finally, we will elicit perspectives exploring the pathways that lead towards or prevent successful recovery from depression.

Participants: We seek to generate a sample consisting of diverse stakeholders that can provide unique perspectives and help constitute an integrated understanding of the phenomena and processes under investigation. We will engage primary stakeholders such as adolescents (those with both a history of depression and those without) and their parents. Secondary stakeholders will be recruited from the socio-ecological systems and institutions that develop and nurture the environments which shape the experiences of the primary stakeholders. These include schoolteachers and counselors, health service providers, social workers, and policymakers. Accordingly, we use a purposeful sampling strategy to solicit key-informants with direct experience and deep insight of the inquiry topics (e.g. adolescents and parents), or due to the strategic positions they occupy in the health and education systems and social services (e.g. social workers or policymakers).[35]

This multi-perspective approach will allow triangulation of the data through comparison of insights between, and across, stakeholder groups. For KIIs, a sample of 12 qualitative interviews is sufficient for identification of themes in relatively homogeneous groups.[39] Only those policymakers who hold relevant roles and expertise will be recruited for KIIs. Each FGD will be comprised of approximately 8-12 participants. In each site the sample will vary according to context-specific priorities. Table 2 presents the various stakeholder groups targeted for KIIs and FGDs.

For peer review only - http://bmjopen.bmj.com/site/about/guidelines.xhtml

Table 2: Key informant interviews & focus group discussion stakeholders in the IDEA Project

Stakeholders	Brazil		Nepal		Nigeria		UK*
	KII	FGD	KII	FGD	KII	FGD	KII
Adolescents	6	1	12			1	
Health service providers	12		12		12		12
Policymakers	6		6		6	1	12
Parents	6	1	6	2		1	12
Researchers & academics							12
School teachers & counselors	12		12		12		12
Social workers	12		12		12		12

*No FGDs to be conducted in the UK

For each stakeholder group indicated in Table 2, relevant inclusion and exclusion criteria apply. Health service providers need to have training and experience working with adolescents and/or mental health. These can include psychiatrists, psychologists, pediatricians, and general physicians. The inclusion of mental health specialists in the IDEA study is foundational, as these stakeholders are pivotal in providing care to depressed adolescents. However, as adolescents often present to primary care with depression, specifically with somatic symptomology, the inclusion of pediatricians and primary healthcare physicians are essential, especially in settings with diminished numbers of, and limited access to, mental health specialists.[40] We will include those social workers who have experience in working with adolescents. For educators, we will only include teachers and school counselors who work with adolescent students and exclude those working with younger children in the school system. Researchers and academics will be recruited based on expertise in adolescent depression. Policymakers will be included based on relevant linkages to the health, education, and social care systems, and the ability to comment meaningfully on the programmatic and policy environments of the country. Finally, to get rich narratives on context-specific lived experience of depression we will recruit adolescents with current or past history of depression. We will also interview adolescents with no history of depression to broaden our understanding of the knowledge and attitudes surrounding depression. Parents of these two subsets of adolescents will be recruited to gain perspectives of parental attitudes towards, and knowledge of depression, and understand its impact on families.

Data collection: The KII and FGD guides will be developed using an iterative process. We will first create an interview guide drawing from our conceptual framework and conduct approximately six preliminary KIIs with different stakeholders, across each site. The data from these initial KIIs will be used to revise the guides for contextual sensitivity. The subsequent KIIs and FGDs will be conducted with these revised guides, with the provision for further edits if subsequent findings indicate it to be necessary. Please refer to Figure 2 for an overview of the data collection and data analysis process.

All data collection procedures and decisions will be collated into memos to preserve an auditable record of methodological decisions. KIIs and FGDs will be audio recorded with consent (and assent where applicable), and audio recordings will be professionally translated to

For peer review only - http://bmjopen.bmj.com/site/about/guidelines.xhtml

English (except brazil, where analysis will be conducted in Portuguese) for data analysis. Local terminology for depression and idioms of distress will be included in the local language along with English translations.

Researchers will complete debriefing forms to capture salient ideas, important exchanges, salient events, and other features of the interaction during KIIs and FGDs. Debriefing forms are an essential component of the data analysis process and are intended to provide meaning and understanding of the culture, social situation, or phenomenon under investigation, and contextualize the interview transcript.[35, 41]

Data analysis: The major deductive themes of the qualitative study are as follows:

- Understanding of developmental, social, and health changes in adolescents
- Understanding of depression in adolescents: symptoms, impact, and help-seeking (e.g., how can depression be detected early and where, whom, and when would be best to identify it early)
- Perception of causes or contributory factors to depression in adolescents
- Views regarding risk detection and possible preventive measures (primary and secondary prevention), including their feasibility, acceptability, and utility

We will use framework analysis (FA) to guide the data collection and analysis of this study[42, 43] following the stages of the FA approach in applied qualitative research:

1. Transcription
2. Familiarization with the interview
3. Coding
4. Developing a working analytical framework
5. Applying the analytical framework
6. Charting data into the framework matrix
7. Interpreting the data

We will adopt a modified approach to implementing the framework approach, as presented in Figure 1. The first step will be to create the KII and FGD interview guides and develop a universal codebook that can be used across the four countries. This deductive codebook will be derived from the study objectives, existing literature, theory, and expert knowledge. Each country will adjust the codebook to include country-specific codes as necessary. The methodological approach to data collection and analysis will be cyclical and iterative, with each step informing and enhancing subsequent steps, as presented in Figure 1.

<insert Figure-2 here>

Figure 2: Data collection and analysis approach.

IDEA researchers across the four country sites will code the data. Codes and themes will be refined using the constant comparison method.[44] This involves moving back and forth between newly coded data and comparing it with previously coded segments to check that the code is still capturing the same essence in the excerpts, as data analysis continues to mature.

For peer review only - http://bmjopen.bmj.com/site/about/guidelines.xhtml

The addition of new codes and themes will continue until no new codes, categories, or themes emerge from the data, indicating theoretical saturation.[45]

Throughout analysis, researchers will maintain memos to capture ideas, themes, problems, etc., that are extracted from the coding process. Inductive themes can be identified at any stage during the analysis. We will engage in a reflexive approach to data analysis, critically reflecting upon the theoretical structures that are drawn out of the analysis.[46] Wherever applicable we will reconceptualize the evidence using other possible theoretical and conceptual models, to test, validate or refine ideas and findings.

Once coding is completed, a summary of each code will be written capturing the essence of that code across the whole dataset, with supporting quotes and researcher insights. We will stratify results according to site and stakeholder characteristics to gain nuanced understandings of how themes vary and converge across groups. In a final step, thematic narratives will be developed in preparation for article manuscripts. For each theme, we will present results from each site to facilitate comparison across countries. We will present similarities and differences in results, across stakeholders and across sites. If applicable, we will construct and present explanatory narratives for any heterogeneity in the results i.e. possibly attributing to cultural or contextual factors. We will use NVivo version-12 software for coding and analysis.[47]

PATIENT AND PUBLIC INVOLVEMENT

Youth advisory boards have been engaged or established in each of the IDEA countries. The overall IDEA study has been discussed with members of the youth advisory board, and they will be regularly updated on the study progress throughout. The dissemination plan will be developed in accord with the youth advisory boards. Youth advisory boards are not responsible for participant recruitment.

ETHICS AND DISSEMINATION

The IDEA qualitative study has been reviewed and approved by the institutional review board of the George Washington University, USA. Additionally, the study has country-specific approvals from the Nepal Health Research Council in Nepal; the Ethics Committee at Hospital de Clínicas de Porto Alegre, in Brazil; the Lagos State University Teaching Hospital Research and Ethics Committee and The Research and Ethics Committee of The Federal Neuropsychiatry Hospital Yaba, Lagos, in Nigeria; and the Psychiatry, Nursing and Midwifery Research Ethics Subcommittee at King's College London, in the UK.

All data collection will operate under the ethical principles of informed consent and assent. Data from the project will be de-identified and stored in password-protected computer servers with access restricted to essential study personnel only. All results will be presented in the aggregate to minimize any potential risks to confidentiality of research participants. Study findings will be reported via publications in academic journals and conferences. In addition to coordination with youth advisory boards, results will also be disseminated through traditional and social media, wherever applicable. We will follow the Consolidated criteria for Reporting Qualitative research (COREQ) guidelines in resultant publications.[48]

DISCUSSION

For peer review only - http://bmjopen.bmj.com/site/about/guidelines.xhtml

In this protocol we discuss a systematic and progressive set of methods to understand the complex problem of contextual variations in identifying depression early in adolescence globally. Through this research we seek to establish the acceptability, feasibility, and utility of integrating such approaches in Brazil, Nepal, Nigeria, and the UK. Approaches determined to be culturally acceptable, feasible, and effective could then be proposed for health providers to support the early detection of depression or identification of risk factors. Identifying depression early in adolescence and understanding the risk and protective factors of depression would inform policy decisions for funding allocation, amendments to legislation, and programming for interventions. Although the prominence of mental health services in health systems varies across countries of different income classifications, compiling stakeholder perspectives can help tailor national mental health policies toward the needs of the specific country. For instance, revelations regarding the effectiveness of existing policies or the level of public awareness about mental illness provide national and community leadership with the context necessary to actualize improvements in current mental health infrastructure.

Investigating these issues using qualitative methods in multiple global settings has not been conducted to date. Therefore, this study will contribute to addressing this gap in the literature. Additionally, there is growing movement and discussion on the transparency, reliability, validity and reproducibility of qualitative research.[49] A systematic presentation of the conceptualization and procedural implementation of such studies can contribute to the discussion surrounding the transparency and veracity of qualitative methods, and its utility for global mental health research.

The variety of stakeholders to be interviewed presents the opportunity for a range of solutions to be developed across sectors, potentially in the form of public campaigns, health governance restructuring, or additions to school resources. Engaging community members and leaders in discussions surrounding adolescent depression is crucial for the sustainability of these changes. By investigating these issues across a range of low-, middle- and high-income settings, we hope to generate context-specific, and potentially global understandings of, and responses to, depression among the world's 1.2 billion adolescents.

Study Status: The IDEA qualitative study has finished primary data collection and is in the process of analyzing data and preparing manuscripts for publication.

REFERENCES

1. Belfer, M.L. Child and adolescent mental disorders: the magnitude of the problem across the globe. *J Child Psychol Psychiatry*, 2008. 49(3): p. 226-36.
2. Thapar, A., Collishaw, S., Pine, D.S., et al. Depression in adolescence. *Lancet*, 2012. 379(9820): p. 1056-67.
3. Avenevoli, S., Swendsen, J., He, J.-P., et al. Major depression in the national comorbidity survey-adolescent supplement: prevalence, correlates, and treatment. *J Am Acad Child Psy*, 2015. 54(1): p. 37-44.e2.
4. Cipriani, A., Zhou, X., Del Giovane, C., et al. Comparative efficacy and tolerability of antidepressants for major depressive disorder in children and adolescents: a network meta-analysis. *The Lancet*, 2016. 388(10047): p. 881-890.

For peer review only - http://bmjopen.bmj.com/site/about/guidelines.xhtml

5. Kieling, C., Adewuya, A., Fisher, H.L., et al. Identifying depression early in adolescence. *Lancet Child Adolesc Health*, 2019. 3(4): p. 211-213.
6. Henrich, J., Heine, S.J. and Norenzayan, A. The weirdest people in the world? *Behav Brain Sci*, 2010. 33(2-3): p. 61-83; discussion 83-135.
7. Ormel, J., Cuijpers, P., Jorm, A.F., et al. Prevention of depression will only succeed when it is structurally embedded and targets big determinants. *World Psychiatry*, 2019. 18(1): p. 111-112.
8. Lindström, J. and Tuomilehto, J. The Diabetes Risk Score. *Diabetes Care*, 2003. 26(3): p. 725.
9. Lloyd-Jones, D.M. Cardiovascular risk prediction: basic concepts, current status, and future directions. *Circulation*, 2010. 121(15): p. 1768-77.
10. Parker, J.S., Mullins, M., Cheang, M.C.U., et al. Supervised risk predictor of breast cancer based on intrinsic subtypes. *J Clin Oncol*, 2009. 27(8): p. 1160-1167.
11. Gautam, K., Pedersen, G.A., Wahid, S.S., et al., Ethical considerations for conducting cross-cultural biological psychiatry and prevention research on depression among adolescents in low- and middle-income countries, in Developments in Neuroethics and Bioethics. 2019, Academic Press.
12. Cuijpers, P., Beekman, A.T. and Reynolds, C.F., 3rd. Preventing depression: a global priority. *JAMA*, 2012. 307(10): p. 1033-4.
13. Botter-Maio Rocha, T., Fisher, H.L., Caye, A., et al. Identifying adolescents at risk for depression: a prediction score performance in cohorts based in three different continents. *J Am Acad Child Adolesc Psychiatry*, 2020. doi: 10.1016/j.jaac.2019.12.004. [Epub ahead of print]
14. Brathwaite, R., Rocha, T.B., Kieling, C., et al. Predicting the risk of depression among adolescents in Nepal using a model developed in Brazil: the IDEA Project. *Eur Child Adolesc Psychiatry*, 2020. doi: 10.1007/s00787-020-01505-8. [Epub ahead of print]
15. McLeroy, K.R., Bibeau, D., Steckler, A., et al. An ecological perspective on health promotion programs. *Health Educ Q*, 1988. 15(4): p. 351-77.
16. Baer, H., Singer, M. and Susser, I., Medical Anthropology and the World System. Third ed. 2013, Santa Barbara, California Praeger.
17. Engel, G.L. The need for a new medical model: a challenge for biomedicine. *Science*, 1977. 196(4286): p. 129.
18. Kleinman, A., Patients and healers in the context of culture. 1980, London, England: University of California Press Ltd.
19. Nichter, M. Idioms of distress: alternatives in the expression of psychosocial distress: a case study from South India. *Cult Med Psychiatry*, 1981. 5(4): p. 379-408.
20. Cairns, K.E., Yap, M.B., Reavley, N.J., et al. Identifying prevention strategies for adolescents to reduce their risk of depression: a Delphi consensus study. *J Affect Disord*, 2015. 183: p. 229-38.
21. Morgan, A.J. and Jorm, A.F. Self-help strategies that are helpful for sub-threshold depression: a Delphi consensus study. *J Affect Disord*, 2009. 115(1-2): p. 196-200.
22. Langlands, R.L., Jorm, A.F., Kelly, C.M., et al. First aid for depression: a Delphi consensus study with consumers, carers and clinicians. *J Affect Disord*, 2008. 105(1-3): p. 157-65.

For peer review only - http://bmjopen.bmj.com/site/about/guidelines.xhtml

23. Yap, M., Pilkington, P., Ryan, S., et al. Parenting strategies for reducing the risk of adolescent depression and anxiety disorders: a Delphi consensus study. *J Affect Disord*, 2014. 156: p. 67-75.
24. Grunder, G., Bauer, M., Deuschle, M., et al. [Rank of outcome parameters in the treatment of depression : Results of a Delphi panel survey]. *Nervenarzt*, 2017. 88(9): p. 1044-1049.
25. Jones, J. and Hunter, D. Consensus methods for medical and health services research. *Bmj*, 1995. 311(7001): p. 376-80.
26. Blow, A.J. and Sprenkle, D.H. Common factors across theories of marriage and family therapy: a modified Delphi study. *J Marital Fam Ther*, 2001. 27(3): p. 385-401.
27. Sori, C.F. and Sprenkle, D.H. Training family therapists to work with children and families: A modified delphi study. *J Marital Fam Ther*, 2004. 30(4): p. 479-495.
28. Murphy, M.K., Black, N.A., Lamping, D.L., et al. Consensus development methods, and their use in clinical guideline development. *Health Technol Assess*, 1998. 2(3): p. i-iv, 1-88.
29. Pedersen, G.A., Zajkowska, Z., Kieling, C., et al. Protocol for a systematic review of the development of depression among adolescents and young adults: psychological, biological, and contextual perspectives around the world. *Syst Rev*, 2019. 8(1): p. 179.
30. Smith, J.J. and Borgatti, S.P. Salience counts—and so does accuracy: correcting and updating a measure for free-list-item salience. *J Linguist Anthropol*, 1997. 7(2): p. 208-209.
31. Qualtrics, Qualtrics. 2019, Qualtrics: Provo, Utah, USA.
32. Braun, V. and Clarke, V. Using thematic analysis in psychology. *Qual Res Psychol*, 2006. 3(2): p. 77-101.
33. De Silva, M.J., Breuer, E., Lee, L., et al. Theory of Change: a theory-driven approach to enhance the Medical Research Council's framework for complex interventions. *Trials*, 2014. 15: p. 267.
34. Vogel, I., Review of the Use of 'Theory of Change' in International Development. 2012, UK Department of International Development.
35. Patton, M.Q., Qualitative Research & Evaluation Methods: Integrating Theory and Practice. 4th ed. 2014, Thousand Oaks, CA: Sage Publications.
36. Remler, D.K. and Van Ryzin, G.G., Research Methods in Practice. 2015, Los Angeles, CA: SAGE.
37. Haroz, E.E., Ritchey, M., Bass, J.K., et al. How is depression experienced around the world? A systematic review of qualitative literature. *Soc Sci Med*, 2017. 183: p. 151-162.
38. Gorman, J.M. Comorbid depression and anxiety spectrum disorders. *Depress Anxiety*, 1996. 4(4): p. 160-8.
39. Guest, G., Bunce, A. and Johnson, L. How many interviews are enough? *Field Methods*, 2006. 18(1): p. 59-82.
40. Mauerhofer, A., Berchtold, A., Michaud, P.A., et al. GPs' role in the detection of psychological problems of young people: a population-based study. *Br J Gen Pract*, 2009. 59(566): p. e308-14.
41. Emerson, R., Fretz, R. and Shaw, L., Writing ethnographic fieldnotes. 1995, Chicago: University of Chicago Press.

For peer review only

For peer review only - http://bmjopen.bmj.com/site/about/guidelines.xhtml

42. Gale, N.K., Heath, G., Cameron, E., et al. Using the framework method for the analysis of qualitative data in multi-disciplinary health research. *BMC Med Res Methodol*, 2013. 13(1): p. 117.
43. Smith, J. and Firth, J. Qualitative data analysis: the framework approach. *Nurse Res*, 2011. 18(2): p. 52-62.
44. Glaser, B.G. and Strauss, A.L., The Discovery of Grounded Theory: Strategies for Qualitative Research. 1967, Chicago: Aldine.
45. Strauss, A. and Corbin, J., Basics of Qualitative Research: Grounded Theory procedures and techniques. 1990, Newbury Park, CA: SAGE.
46. Finlay, L. Negotiating the swamp: the opportunity and challenge of reflexivity in research practice. *Qual Res*, 2002. 2(2): p. 209-230.
47. Richards, L., Using NVivo in qualitative research. 1999, London, England: Sage Publications.
48. Tong, A., Sainsbury, P. and Craig, J. Consolidated criteria for reporting qualitative research (COREQ): a 32-item checklist for interviews and focus groups. *Int J Qual Health C*, 2007. 19(6): p. 349-357.
49. Tsai, A.C., Kohrt, B.A., Matthews, L.T., et al. Promises and pitfalls of data sharing in qualitative research. *Soc Sci Med*, 2016. 169: p. 191-198.

For peer review only

For peer review only - http://bmjopen.bmj.com/site/about/guidelines.xhtml

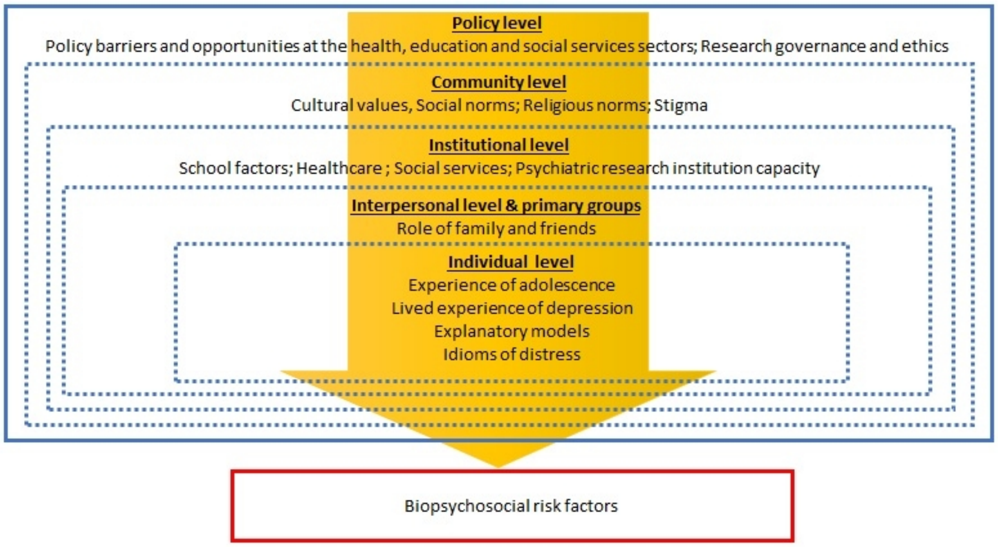

Figure 1: Conceptual framework for the qualitative component of the IDEA study.

164x90mm (120 x 120 DPI)

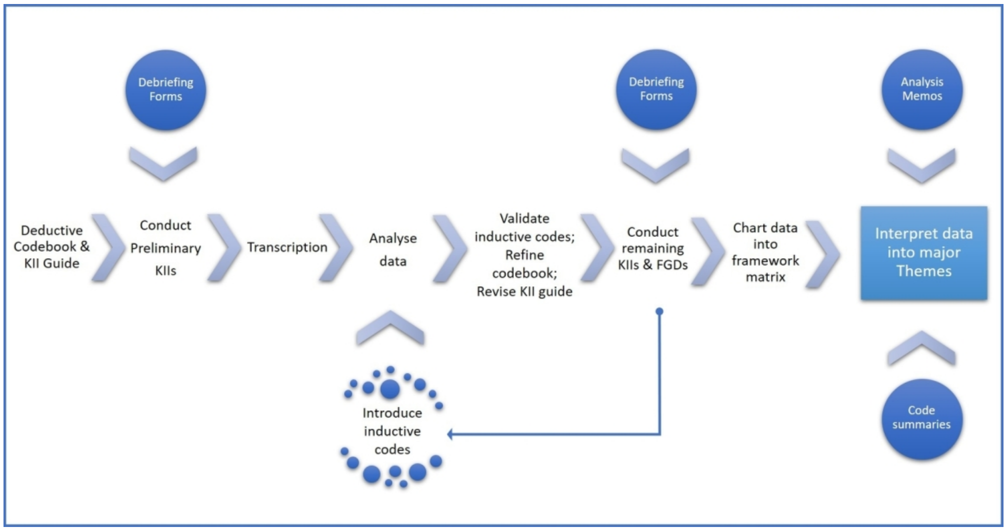

Figure 2: Data collection and analysis approach.

138x72mm (300 x 300 DPI)

Appendix-1

Study settings: Country descriptions

Brazil

Brazil is an upper middle-income country representing the growing number of countries with rapidly developing economies and urbanization, collectively known as the BRICS nations (Brazil, Russia, India, China, and South Africa). Two out of every three adolescents worldwide live in a BRICS country. Brazil has a total population of 210 million people; and the prevalence of major depressive disorder has been estimated to be around 9.4 percent among adults, being one of the highest rates in Latin America.[1] The health and education systems are characterized by a mix of public and private services. Overall, the education system is divided into preschool, fundamental school (nine years) and high school (three additional years). The public Unified Health System provides universal health coverage free of charge. Mental health care is provided at multiple levels of complexity, including psychosocial centers (2,462 units in 2017, with only 229 specialized to the care of children and adolescents).[2] The social services system is organized based on the specificities of each territory. The child care council is the organization responsible for ensuring the rights of children and adolescents.[3]

In Brazil, the qualitative study will be implemented by a team from the Universidade Federal do Rio Grande do Sul. Data collection efforts will be led by five researchers from IDEA Brazil team: one psychologist, two psychology undergraduate students, and one with social communication training (all females); and two medical doctors (both males), which includes an IDEA principal investigator. The recruitment process will utilize connections of the IDEA Brazil team to local, relevant, institutions. The key informant interviews (KII) will take place in private and convenient locations for each participant, and the focus group discussions (FGD) will be held in Hospital de Clínicas de Porto Alegre.

Nepal

Nepal is one of the poorest countries in the world and represents conditions of adolescents living in the least-developed nations. Nepal also is representative of the large number of adolescents living in humanitarian settings. Adolescents in Nepal today grew up during a decade-long civil war, and many have been impacted by repeated environmental disasters including earthquakes, flooding, and landslides. More than 20 percent of the national population have at least one mental health problem in Nepal.[4] Adolescents comprise 24 percent of the total population of Nepal, and 14 percent of this population have one or more psychosocial problems.[5, 6] Known risk factors contributing to depression include political conflict, caste-based disparities, earthquake, urban habitation, domestic violence, poverty, and other social factors.[7-9] There is a single outpatient child and adolescent psychiatry clinic and no corresponding inpatient facilities. The National Mental Health Policy was developed in Nepal in 1996 to integrate mental health into primary health care services.[10] The Ministry of Health and Population has adapted the World Health Organization’s (WHO) Mental Health Gap Action Programme (mhGAP), developed a community mental health care package training curricula to non-specialized mental health service providers, revised psychotropic medications in the

For peer review only

essential drug list, included four priority Mental Neurological and Substance use disorders (depression, psychosis, epilepsy and alcohol use disorder) in basic health service package and revised the National Adolescent Health and Development Strategy.[11] The Ministry of Health & Population has been drafting the National Mental Health Strategy and Action Plan in an attempt to reinforce integration of mental health into primary care with a focused strategy on child and adolescent mental health which was not included in the National Mental Health Policy 1996. The Ministry of Education has piloted the introduction of school nurses as an initiative to improve child and adolescent health in selected regions of the country.

The data collection efforts in Nepal will be implemented by the Transcultural Psychosocial Organization Nepal (TPO Nepal), one of Nepal's leading psychosocial organizations for providing clinical care and conducting mental health research. TPO interviewers and FGD facilitators will include two female researchers with graduate level training in Public Health and one male researcher with graduate training in management studies, under the guidance of a senior TPO director and researcher, who is an IDEA study Co-investigator. Recruitment for the study will be facilitated by reaching out to institutions offering adolescent mental health services in Kathmandu district in Nepal. KIIs will be conducted in private locations at health facilities, schools, stakeholder offices, TPO Nepal offices, Ministry offices, etc. The FGDs will each be conducted at TPO offices. KIIs and FGDs will be primarily conducted in Nepali & English.

Nigeria

Nigeria is a lower-income country and the most populous country in Africa. The experiences of adolescents in Nigeria reflect the rapid development of African economies as well as chronic exposure to political violence, community violence, and high rates of infectious diseases, including HIV/AIDS. In Nigeria, adolescents (age 10-19 years) make up approximately 41 million, or 23 percent of the total population of 180 million.[12] In 2015, the WHO estimated the prevalence of depression to be approximately 3.9 percent in the general population.[13] However, nationally representative research has indicated that up to 10 percent of the Nigerian school adolescents to have clinical depression.[14] With over 15 million adolescents in its secondary schools, Nigeria has an estimated 1.5 million depressed school adolescents in need of intervention.[15] Mental health remains under-prioritized, and is unaddressed in the National Health Policy and National Strategic Health Development Plan (2010-2015), as well as in educational and criminal justice policies.[16] Similarly, child and adolescent mental health are not mentioned in the National Child Health Policy. Additionally, the School Health Policy launched by the Federal Government in 2006 did not have provisions for mental health.[17] There are no government-run health facilities specifically for children and adolescents, and less than 20 percent of primary and secondary schools have activities that promote mental health.[18] Social and health risk factors for poor mental health, particularly for children and adolescents, include high levels of poverty and child malnutrition, as well as the prevalence of child labor and under-age marriage.[19] In Lagos State, where the IDEA project will be implemented, there are over one million adolescents in its nearly 3,000 secondary schools.[20]

In Nigeria, the study is managed by the Lagos State University, College of Medicine, Department of Behavioral Medicine. Data collection efforts will be led by a team of two female doctors, a consultant psychiatrist, and a medical officer under guidance of a senior professor, who is an IDEA project co-investigator. Recruitment will be through flyers distributed to social

For peer review only

workers, health care providers and the teachers in both public and private schools in Lagos. Letters will be written to the permanent secretaries of the ministries of health, education, social services and the health service commission requesting the nomination of civil servants for policy-maker interviews of the respective ministries. Interviews will be done at the convenience of the participant, and the timing and the location will be flexible. Interviews can be held at the office of the researchers or at the offices of the person being interviewed. The FGDs will be held at Department of Behavioural Medicine at The Lagos State University Teaching Hospital and at the Child and Adolescent Mental Health Service of The Federal Neuropsychiatry Hospital Oshodi Annex.

United Kingdom

The UK is the study setting representative of adolescents living in highly resourced regions such as Western Europe and North America. Adolescents aged 10-19 years old comprise approximately 12 percent of the total population of the UK, of whom 17 percent (2.3 million children) are living in persistent poverty, and 18 percent do not attain a baseline level of proficiency in reading to enable them to participate effectively in activities requiring literacy.[21-23] In the UK, one in seven 11-16 year-olds has at least one mental illness, however, only 25 percent of these receive support from the National Health Service (NHS).[24] The NHS Long Term Plan[25] highlights prevention as a national priority for mental health. Members of the UK also have their own mental health policies, strategies, or programs for the general population. England, Scotland, and Wales have national mental health strategies and policies that support those with mental illnesses by promoting anti-stigma campaigns and recommendations for health providers. In the UK, Child and Adolescent Mental Health Services (CAMHS) are provided by the NHS and cover community and hospital-based care up to 17 years of age. Countries within the UK each feature some variant of a task force or program responsible for improving the quality and accessibility of health care for children and young people, aiming to increase government funding, decrease waiting times, improve school counseling services, and increase social services provision to support child development.[24, 26]

In the UK, the data collection efforts are implemented by King's College London. The primary data collector is a female social scientist with graduate training in nursing and psychology, with experience in conducting qualitative research. The UK activities are guided by a co-investigator of the IDEA project. Participants will be recruited using an opportunistic sampling method, with stakeholder specific adverts being circulated via professional networks, social media, and placed in key locations in the community, including local NHS hospitals. The KIIs will take place at either one of the King's College London campuses, stakeholder's office spaces where appropriate, or over the telephone.

References

1. Andrade, L.H., Wang, Y.P., Andreoni, S., et al. Mental disorders in megacities: findings from the Sao Paulo megacity mental health survey, Brazil. *PLoS One*, 2012. 7(2): p. e31879.
2. Trape, T.L. and Campos, R.O. The mental health care model in Brazil: analyses of the funding, governance processes, and mechanisms of assessment. *Rev Saude Publica*, 2017. 51(0): p. 19.

For peer review only

3. Ministry of Social Development and Fight Against Hunger. 2011.
4. Risal, A., Manandhar, K., Linde, M., et al. Anxiety and depression in Nepal: prevalence, comorbidity and associations. *BMC Psychiatry*, 2016. 16(1): p. 102.
5. Adhikari R.P., Upadhaya N., Pokhrel R., et al. Health and social vulnerability of adolescents in Nepal. *SM J Public Health and Epi*, 2016. 2(3): p. 1032.
6. Central Bureau of Statistics, National Population and Housing Census 2011,. 2011, Government of Nepal: Kathmandu, Nepal.
7. Upadhaya, N., Tize, C., Adhikari, R., et al. Geographies of adolescent distress: A need for a community-based psychosocial care system in Nepal. *Intervention*, 2018. 17.
8. Kohrt, B.A., Hruschka, D.J., Worthman, C.M., et al. Political violence and mental health in Nepal: prospective study. *Br J Psychiatry*, 2012. 201(4): p. 268-75.
9. Kohrt, B.A., Jordans, M.J.D., Tol, W.A., et al. Social ecology of child soldiers: child, family, and community determinants of mental health, psychosocial well-being, and reintegration in Nepal. *Transcult Psychiatry*, 2010. 47(5): p. 727-753.
10. Luitel, N.P., Jordans, M.J., Adhikari, A., et al. Mental health care in Nepal: current situation and challenges for development of a district mental health care plan. *Confl health*, 2015. 9: p. 3-3.
11. Kohrt, B.A., Mutamba, B.B., Luitel, N.P., et al. How competent are non-specialists trained to integrate mental health services in primary care? Global health perspectives from Uganda, Liberia, and Nepal. *Int Rev Psychiatry*, 2018. 30(6): p. 182-198.
12. Affairs, U.N.D.o.E.a.S., World Population Prospects 2019.
13. World Health Organization, Depression and other common mental disorders: Global health estimates. 2017, World Health Organization: Geneva.
14. Adewuya, A.O., Ola, B.A. and Aloba, O.O. Prevalence of major depressive disorders and a validation of the Beck Depression Inventory among Nigerian adolescents. *Eur Child Adolesc Psychiatry*, 2007. 16(5): p. 287-92.
15. National Population Commission (NPC) [Nigeria] and ICF International, Nigeria Demographic and Health Survey 2013. 2014, NPC and ICF International: Abuja, Nigeria, and Rockville, Maryland.
16. Abdulmalik, J., Kola, L. and Gureje, O. Mental health system governance in Nigeria: challenges, opportunities and strategies for improvement. *Global Mental Health*, 2016. 3: p. e9-e9.
17. Federal Ministry of Education Nigeria, National School Health Policy. 2006: Abuja, Nigeria.
18. World health Organization, M.o.H.N., WHO-AIMS report on Mental Health System in Nigeria,. 2006: Ibadan, Nigeria.
19. Atilola, O., Ayinde, O.O., Emedoh, C.T., et al. State of the Nigerian child - neglect of child and adolescent mental health: a review. *Paediatr Int Child Health*, 2015. 35(2): p. 135-43.
20. Education Sector Support Programme in Nigeria (ESSPIN), Lagos Private School Census 2010-2011 Report. 2011, ESSPIN: Lagos, Nigeria.
21. Hagell, A., Coleman, J. and Brooks, F., Key Data on Adolescence 2015. 2015, Association for Young People's Health: London.
22. Social Metrics Commission, Measuring poverty 2019. 2019: United Kingdom.
23. Organization for Economic Cooperation and Development, United Kingdom Country Note – Results from PISA 2015. 2019.
24. National Health Service, Mental Health of Children and Young People in England. 2018.
25. National Health Service, The NHS Long Term Plan. 2019.
26. Health and Social Care, Children and Young People's Mental Health Taskforce: Delivery Plan. 2018, Government of Scotland.

For peer review only
